# Supplementary material for: Solvent-free selective hydrogenation of nitroaromatics to azoxy compounds over Co single atoms decorated on Nb2O5 nanomeshes
Source: Nat Commun. 2024 Apr 12;15:3195. doi: 10.1038/s41467-024-47402-5 (PMC11015025; doi:10.1038/s41467-024-47402-5)
Supplement: Supplementary file 1 — Supplementary Information [file 41467_2024_47402_MOESM1_ESM.pdf]

Supplementary Information

Solvent-free selective hydrogenation of nitroaromatics to azoxy compounds over Co single atoms decorated on Nb_2O_5 nanomeshes

Zhijun Li[1,*] Xiaowen Lu,[1,†] Cong Guo,[2,†] Siqi Ji,[1] Hongxue Liu,[1] Chunmin Guo,[1] Xue Lu,[1] Chao Wang,[3] Wensheng Yan,[3] Bingyu Liu,[4] Wei Wu,[4] J. Hugh Horton,[1,5] Shixuan Xin,[1] Yu Wang[2,*]

[1] National Key Laboratory of Continental Shale Oil, College of Chemistry and Chemical Engineering, Northeast Petroleum University, Daqing, 163318, PR China

[2] Jiangsu Collaborative Innovation Centre of Biomedical Functional Materials, School of Chemistry and Materials Science, Nanjing Normal University, Nanjing 210023, PR China

[3] National Synchrotron Radiation Laboratory, University of Science and Technology of China, Hefei, 230029, PR China

[4] National Center for International Research on Catalytic Technology, School of Chemistry and Material Sciences, Heilongjiang University, Harbin 150080, PR China

[5] Department of Chemistry, Queen′s University, Kingston, K7L 3N6, Canada

† These authors contributed equally: Xiaowen Lu, Cong Guo.

*Corresponding Authors

E-mail: zhijun.li@queensu.ca; yu.wang@njnu.edu.cn;

Chemicals

All chemicals were used as received without further purification. Cobalt(II) acetate tetrahydrate, ammonium niobate(V) oxalate hydrate, melamine, nitrobenzene, aniline, tetrahydrofuran, ethanol, toluene, acetonitrile, isopropanol, sodium hydroxide， hydrogen peroxide (30%), cobalt phthalocyanine, cobalt nitrate hexahydrate, 1-bromo-4-nitrobenzene, 1-fluoro-4-nitrobenzene, 1-iodo-4-nitrobenzene, p-nitrophenol, 4-nitroaniline, p-nitrotoluene, 4-nitroanisole, 3-bromonitrobenzene, 1-chloro-3-nitrobenzene, 3-fluoronitrobenzene, m-nitrotoluene, m-dinitrobenzene, 1-bromo-2-nitrobenzene, 1,2-Dinitrobenzene, o-nitrotoluene, o-chloronitrobenzene, 1-fluoro-2-nitrobenzene, 2-nitroanisole, magnesium chloride, benzoic acid, ammonium metavanadate, oxalic acid, methanol, zinc nitrate hexahydrate, and 2-methylimidazole were purchased from Aladdin. Ammonium chloride, cobalt chloride hexahydrate, 1-chloro-4-nitrobenzene, 3-nitroanisole, m-nitroaniline, and 2-nitroaniline were obtained from Macklin. Deionized water was used throughout this study.

Methods

Synthesis of Co_1/MgO

The synthetic method of MgO was based on a previously reported study[1]. In a typical synthesis, MgO nanosheets with highly exposed (111) facets were prepared with hydrothermal method: 2.0 g of $MgCl_2$ and 0.12 g of benzoic acid were dissolved in 60 ml of deionized water under sonication and then stirred for 15 min. 20 ml of 2 M NaOH solution was added into the mixture with vigorous stirring. The slurry was subsequently transferred to a 100 ml of autoclave and heated at 180 ºC for 24 h. The obtained $Mg(OH)_2$ precursor was washed and dried at 80 ºC. Finally, the MgO(111) nanosheets were obtained following calcination of the $Mg(OH)_2$ precursor in air at 500 ºC. The preparation method for Co_1/MgO was the same as that of Co_1/Nb_2O_5.

Synthesis of Co_1/V_2O_5

The synthetic method of V_2O_5 was based on a previously reported study[2]. In a typical synthesis, 2 g of ammonium metavanadate and 4.5 g of oxalic acid were dispersed in 20 ml of deionized water. Then, the solution was dried by a rotary evaporator and the

obtained powder was calcined at 300 ºC for 4 h to obtain yellow V_2O_5. The preparation method for Co_1/V_2O_5 was the same as that of Co_1/Nb_2O_5.

Synthesis of Co_1/N-C

In a typical synthesis, 0.558 g of $Zn(NO_3)_2·6H_2O$ was dissolved in 15 ml of methanol and subsequently added to 15 ml of methanol with 0.616 g of 2-methylimidazole under sonication. The mixture was left static at room temperature for 12 h before the precipitates were washed and vacuum dried at 80 ºC. The as-prepared ZIF-8 powder was grounded and heated at 950 ºC for 2 h with a ramping rate of 5 ºC min^{-1} to give N-doped carbon (N-C). The preparation method for Co_1/N-C was the same as that of Co_1/Nb_2O_5.

Characterization

X-ray diffraction (XRD) patterns were obtained by using a SmartLab SE diffractometer with Cu Kα radiation. Scanning electron microscopy (SEM) images were taken by an FEI Quanta450 microscope. Transmission electron microscopy (TEM) images were recorded on an FEI Tecnai G2 F20 microscope. High-angle annular dark-field scanning transmission electron microscopy (HAADF-STEM) images were collected by a JEOL JEM-ARM200F TEM/STEM with a spherical aberration corrector. X-ray photoelectron spectroscopy (XPS) measurements were carried out on a Thermo ESCA Lab 250XI spectrometer with a monochromated Al K a radiation (1486.6 eV) source. N_2 sorption analysis were conducted with an ASAP 2460 instrument. Raman spectra were recorded using a Horiba HR Evolution Raman spectrometer with a 532 nm laser. CO chemisorption, H_2-temperature-programmed reduction (H_2-TPR), and H_2-temperature-programmed desorption (H_2-TPD) were performed on a Micromeritics AutoChem II 2920 apparatus. Electron paramagnetic resonance (EPR) spectra were collected with a Bruker EMX plus model spectrometer. Fourier transform infrared (FT-IR) spectroscopy was performed with a Nicolet IS5 spectrometer. In situ CO-DRIFTS were recorded on a Nicolet IS50 FT-IR spectrometer equipped with a liquid nitrogen-cooling mercury-cadmium-telluride (MCT) detector. Before the experiment, the catalyst was pretreated under a high-purity N_2 atmosphere at 130 ºC for 30 min. After

cooling down to room temperature, the background was collected. For the CO adsorption, high-purity CO gas (50 mL/min) was fed into the cell for 30 min up to adsorption saturation. For the CO desorption, the high-purity N_2 flow (30 mL/min) was purged into the cell to remove adsorbed CO. For the nitrobenzene hydrogenation reaction, the procedures were as follows: first, the catalyst was placed in an in-situ reaction cell and pretreated with high purity N_2 flow (30 mL/min) at 130 ºC for 1 h. After cooling down to room temperature, the mixture of gasified nitrobenzene and high-purity N_2 flow (30 mL/min) was introduced into the reaction cell for 30 min, and then the gas flow was changed to H_2/N_2 flow (v: v = 5: 95; 30 mL/min) for nitrobenzene hydrogenation. The metal loadings were analyzed with a PerkinElmer Optima 7300 DV. Thermogravimetric analysis (TGA) was performed on a Setaram Setline STA thermogravimetric analyzer. H_2-D_2 isotopic exchange experiments were conducted on a chemisorption analyzer (Micrometritics, AutoChem II 2920). Specifically, 50 mg of the catalyst was pre-treated in H_2 atmosphere (10 mL/min) at 200 ºC for 1 h and cooled down to 50 ºC. Subsequently, the gas mixture of H_2 (20 mL/min) and D_2 (20 mL/min) was fed into the reactor and the catalyst was heated at a rate of 10 ºC/min. The signals of H_2 (m/z=2), D_2 (m/z=4), and HD (m/z=3) were collected by a mass spectrometer (GSD350 OmniStar) and normalized by the mass of catalyst. X-ray absorption fine structure (XAFS) at Co K-edge of samples were collected in transmission mode at 1W1B beamline of the Beijing Synchrotron Radiation Facility. The EXAFS data were processed using the ATHENA module implemented in the IFEFFIT software packages. To obtain the quantitative structural parameters around central atoms, least squares curve parameter fitting was performed using the ARTEMIS module of IFEFFIT software packages. X-ray absorption near-edge structure (XANES, Co L-edge and O K-edge) measurements were conducted at the Beamline of MCD-A and MCD-B (Soochow Beamline for Energy Materials) and the ultraviolet photoemission spectroscopy (UPS) measurements were performed at the Beamline of BL11U of National Synchrotron Radiation Laboratory in Hefei, China. The valence band spectra were collected with a photon energy of 40 eV and referenced to the Fermi level determined by an Au foil.

Computational methods.

All calculations were performed by using the density functional theory (DFT), as implemented in the Vienna ab-initio simulation package (VASP)[3,4]. The exchange-correlation potential was treated by generalized gradient approximation (GGA) in the form of Perdew-Burke-Ernzerhof (PBE), and the energy cutoff was set to 400 eV[5,6]. All structures were relaxed until a residual force was less than 0.05 eV $Å^{-1}$. The energy convergence criterion was set to 5×10^{-5} eV. The Brillouin zone integration was conducted by using a 2 × 2 × 1 k-point mesh for the calculations of electronic structures and reaction energies. The transition state (TS) was determined using the double-ended surface walking (DESW) and constrained Broyden dimer (CBD) approaches, as implemented in the LASP software[7,8]; the methods can establish a low-energy pathway linking two minima even without iterative optimization of the pathway, from which the TS can be located readily. All transition states were verified by vibrational frequency calculations (only one imaginary frequency). The solvent effect was considered using the Poissson-Boltzmann implicit solvation model[9], in which the dielectric constant ε was taken as 18.36 for THF: H_2O (v: v = 4: 1) as a demonstration[10]. The DFT-D3 method is adopted to correct van der Waals interactions[11]. A vacuum layer in the z-direction was set to over 20 Å. To minimize the self-interaction error, the Hubbard U correction was applied to the 3d orbitals of Co, where the Ueff value was set to 3.4 eV[12].

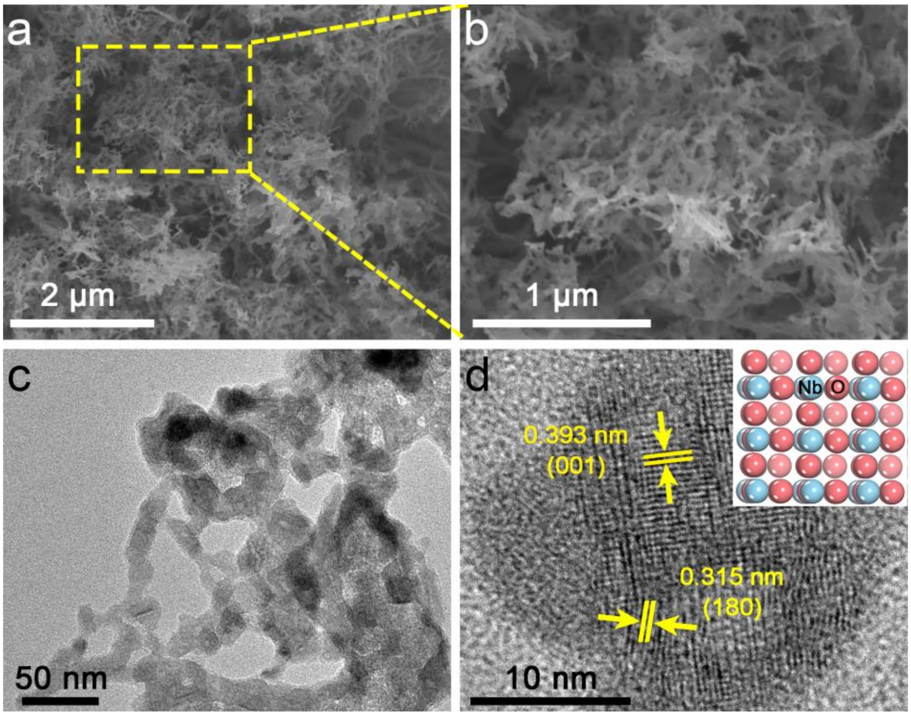

Supplementary Fig. 1 (a,b) SEM, (c) TEM, and (d) HR-TEM images of as-synthesized Nb_2O_5.

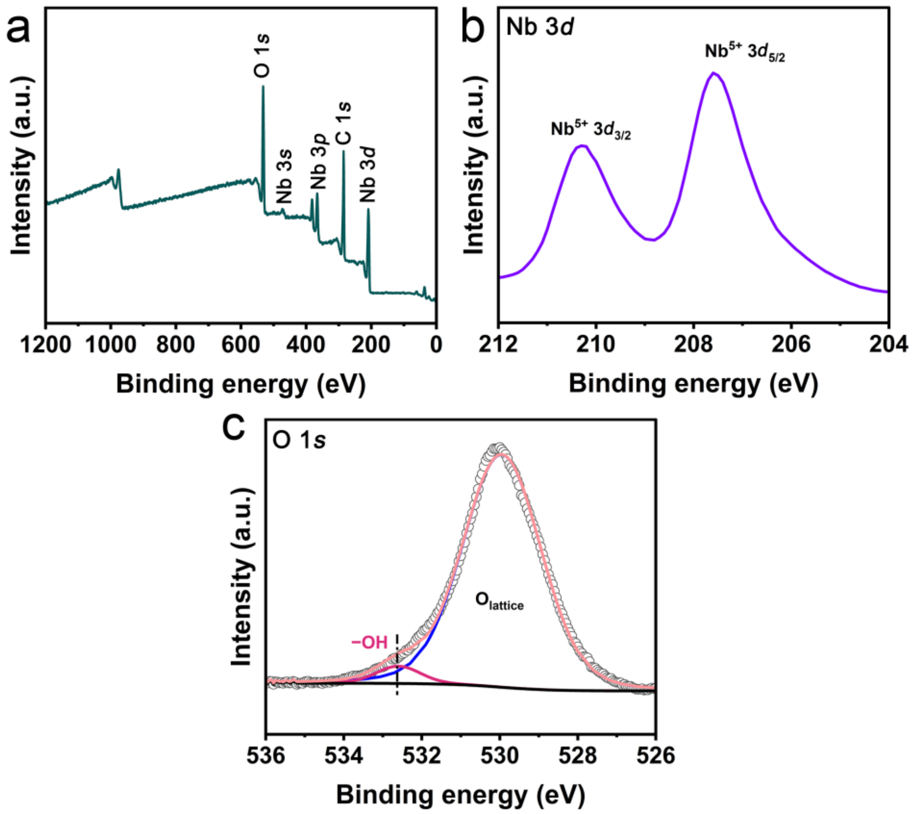

Supplementary Fig. 2 XPS characterization of the as-synthesized Nb_2O_5. (a) The survey scan. High-resolution (b) Nb 3*d* and (c) O 1*s* spectra.

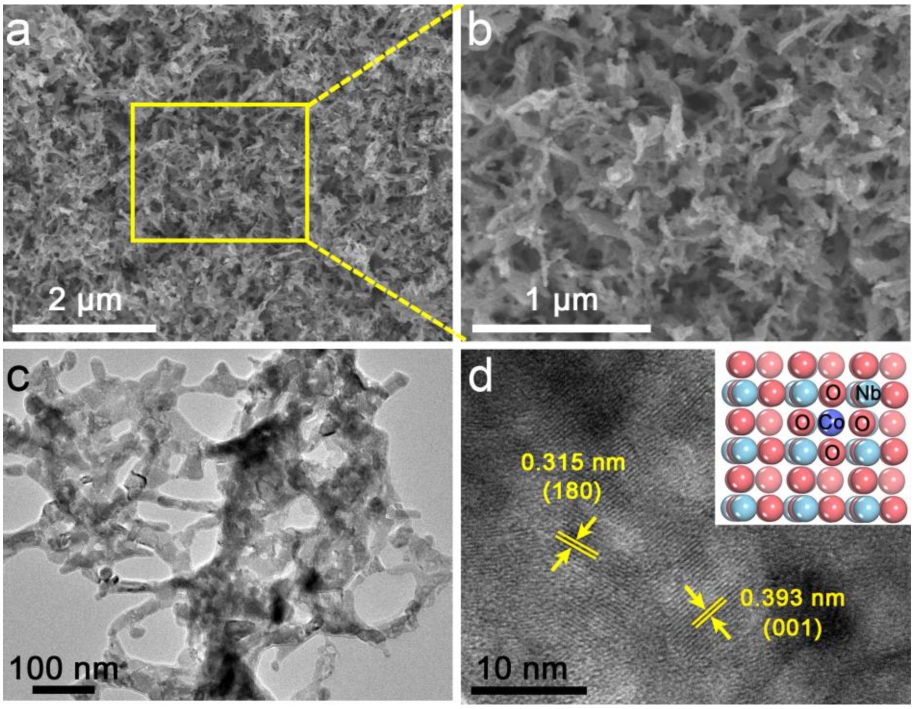

Supplementary Fig. 3 (a,b) SEM, (c) TEM, and (d) HR-TEM images of as-synthesized Co_1/Nb_2O_5. The red, blue, and purple atoms represent O, Nb, and Co, respectively.

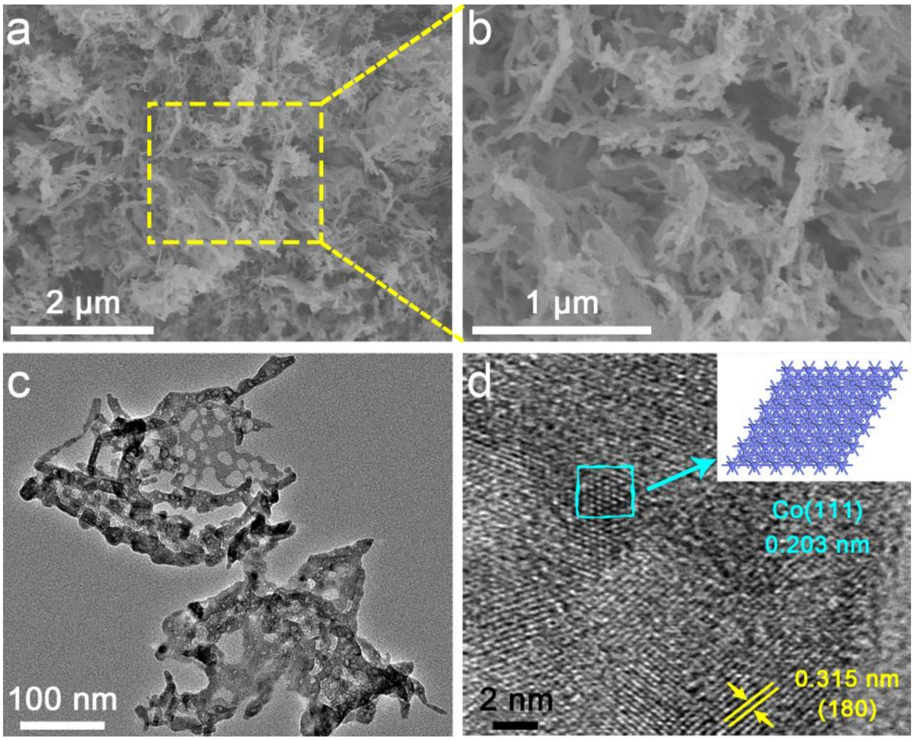

Supplementary Fig. 4 (a,b) SEM, (c) TEM, and (d) HR-TEM images of as-synthesized Co NPs/Nb_2O_5.

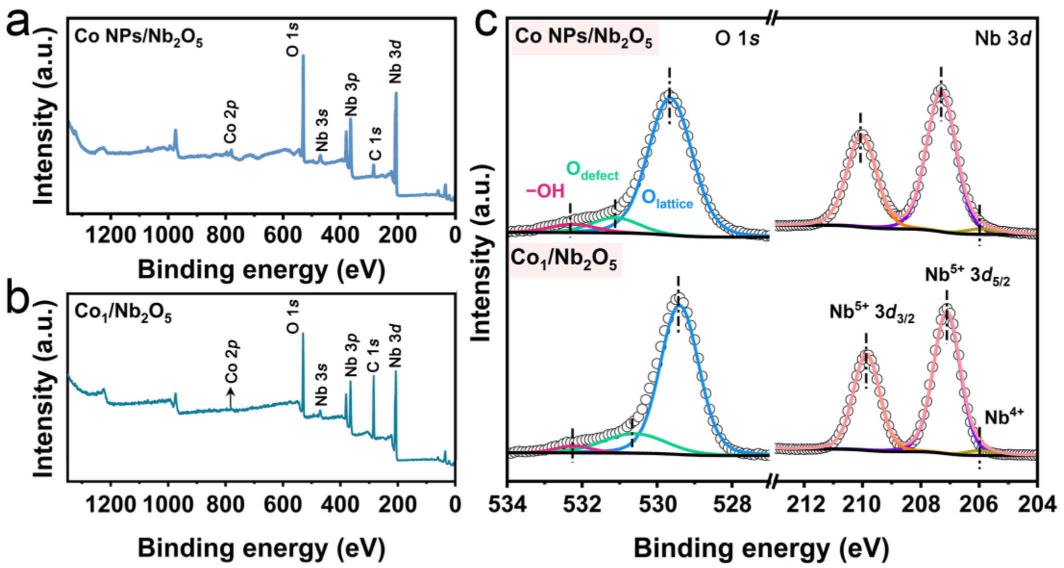

Supplementary Fig. 5 XPS characterization of the as-synthesized Co_1/Nb_2O_5 and Co NPs/Nb_2O_5. (a,b) The survey scan. High-resolution (c) Nb 3*d* and O 1*s* spectra.

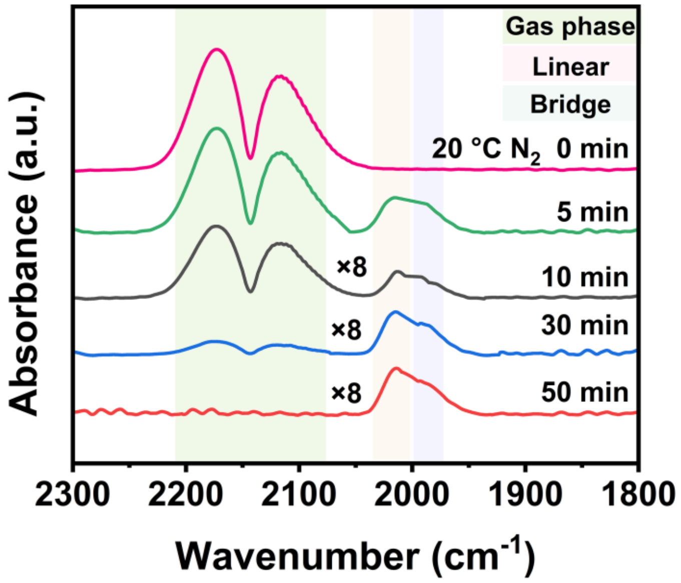

Supplementary Fig. 6 In situ CO-DRIFTS of Co NPs/Nb_2O_5.

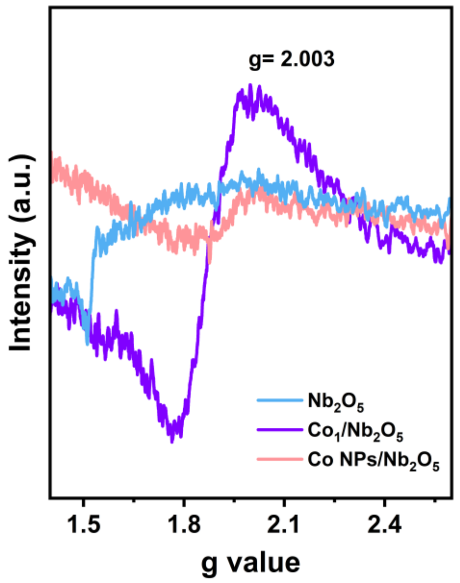

Supplementary Fig. 7 EPR spectra of Nb_2O_5, Co_1/Nb_2O_5, and Co NPs/Nb_2O_5.

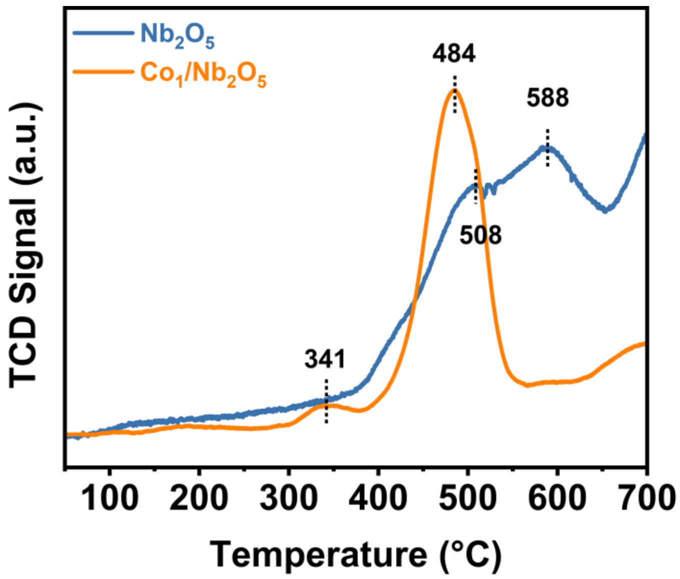

Supplementary Fig. 8 H_2-TPR spectra of Nb_2O_5 and Co_1/Nb_2O_5.

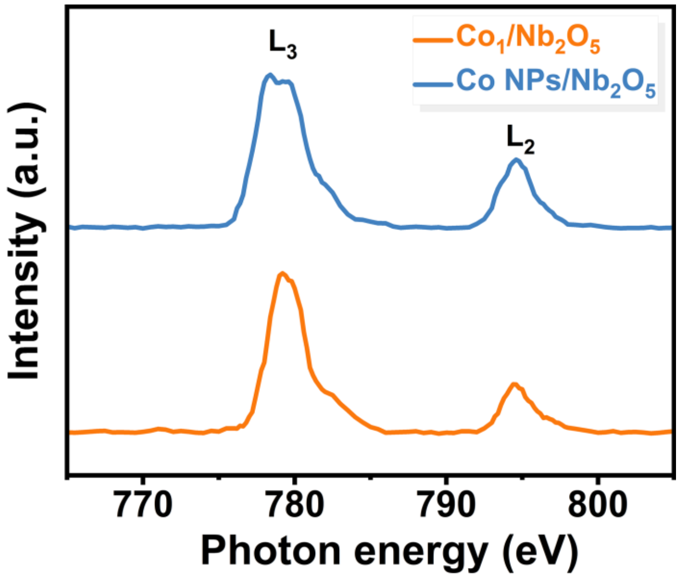

Supplementary Fig. 9 Co L-edge spectra of Co_1/Nb_2O_5, and Co NPs/Nb_2O_5.

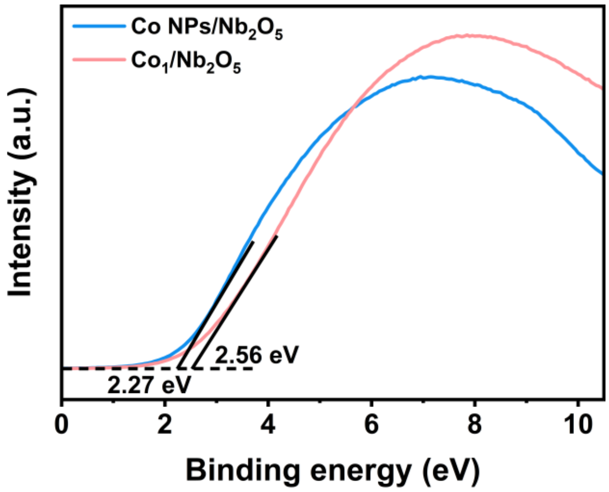

Supplementary Fig. 10 UPS valence band spectra of Co_1/Nb_2O_5 and Co NPs/Nb_2O_5.

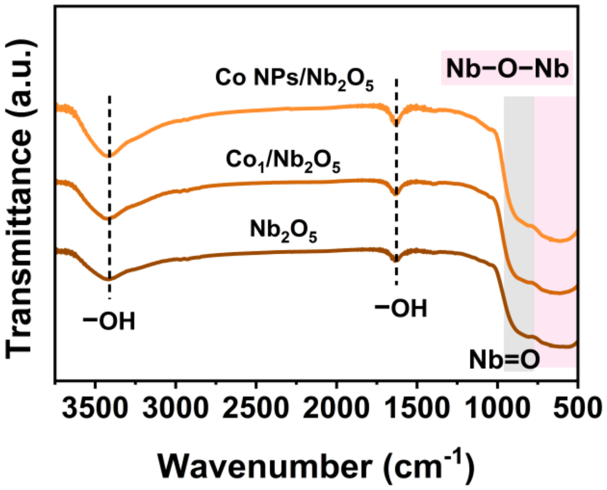

Supplementary Fig. 11 FT-IR spectra of Nb_2O_5, Co_1/Nb_2O_5, and Co NPs/Nb_2O_5.

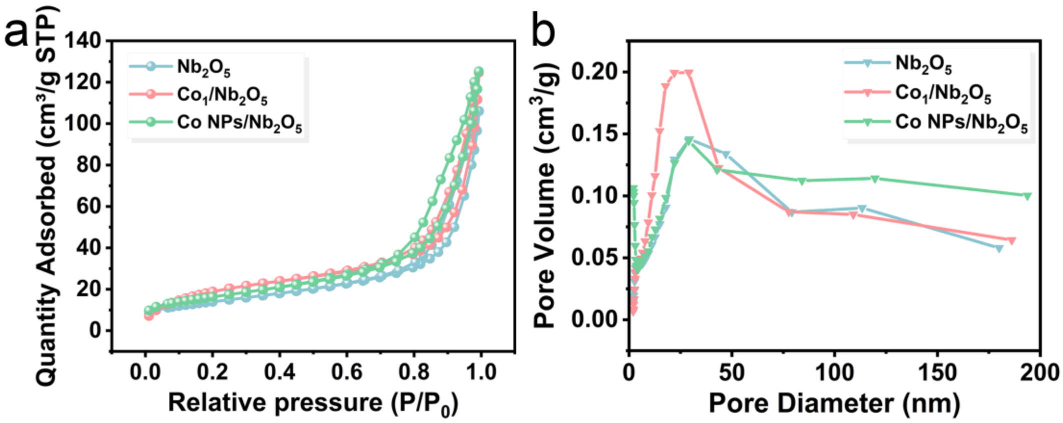

Supplementary Fig. 12 (a) N_2 adsorption/desorption isotherms of Nb_2O_5, Co_1/Nb_2O_5, and Co NPs/Nb_2O_5. (b) The corresponding pore size distributions.

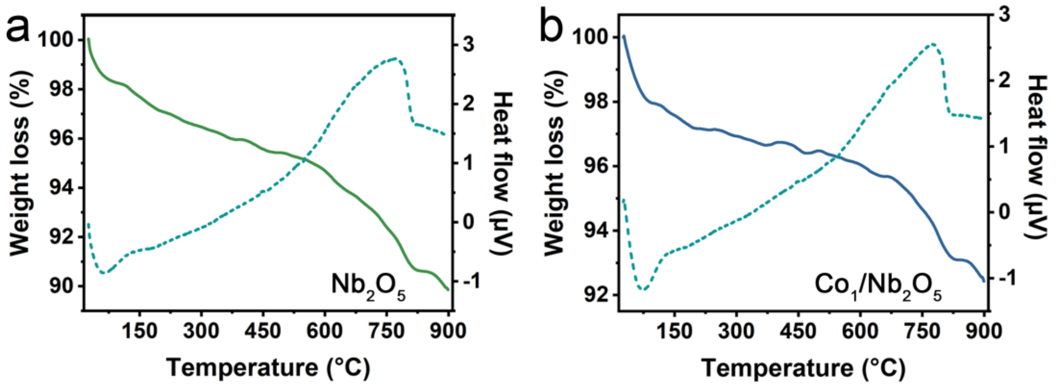

Supplementary Fig. 13 TGA results of (a) Nb_2O_5 and (b) Co_1/Nb_2O_5.

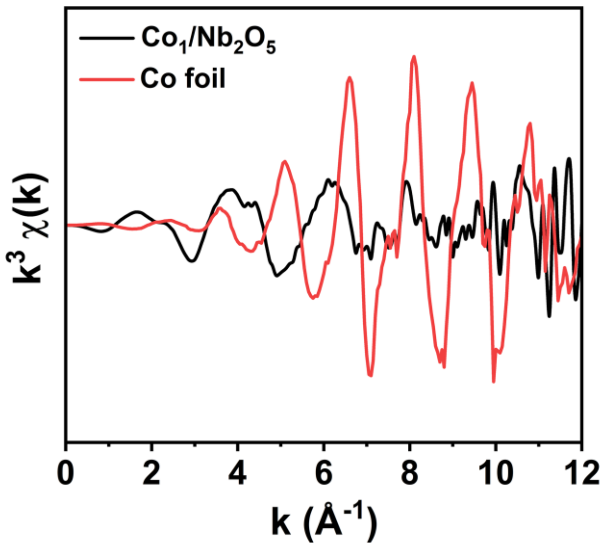

Supplementary Fig. 14 K^3-weighted EXAFS functions at Co K-edge of Co_1/Nb_2O_5 and Co foil.

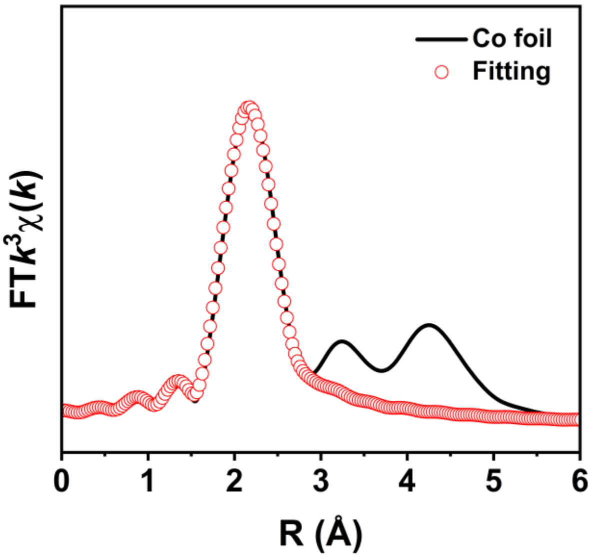

Supplementary Fig. 15 EXAFS fitting in R space of Co foil.

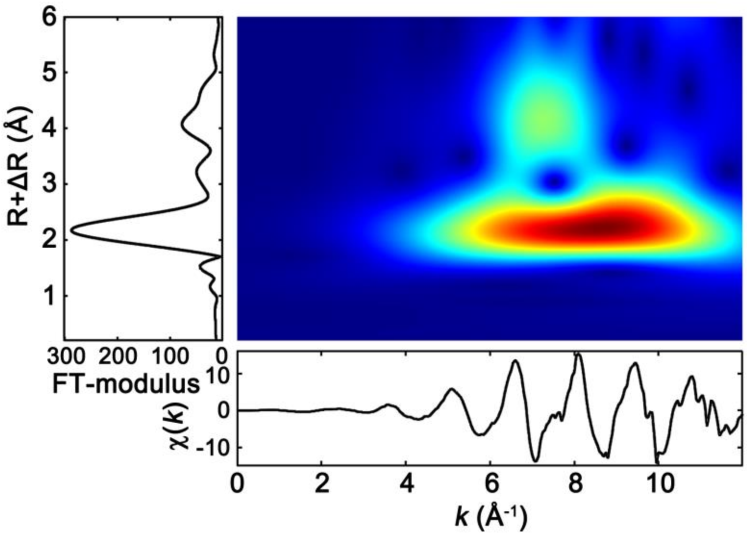

Supplementary Fig. 16 WT-EXAFS of the Co foil.

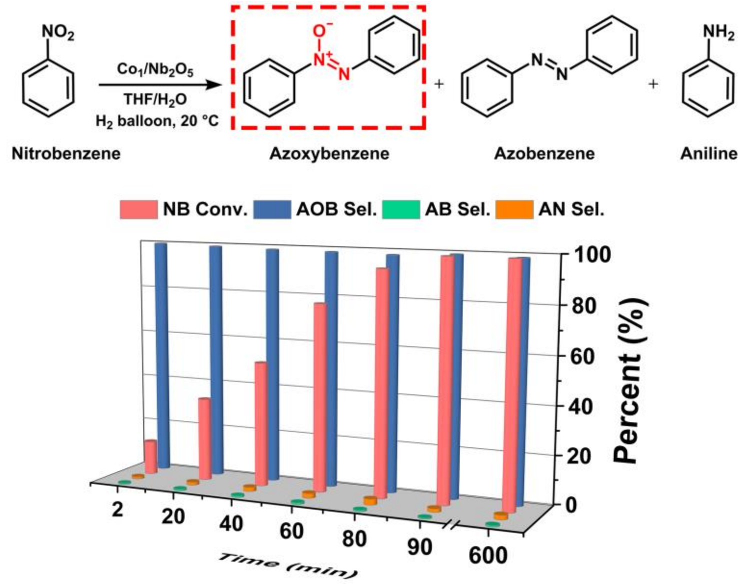

Supplementary Fig. 17 Catalytic performance of Co_1/Nb_2O_5 in the selective hydrogenation of nitrobenzene to azoxybenzene with solvent.

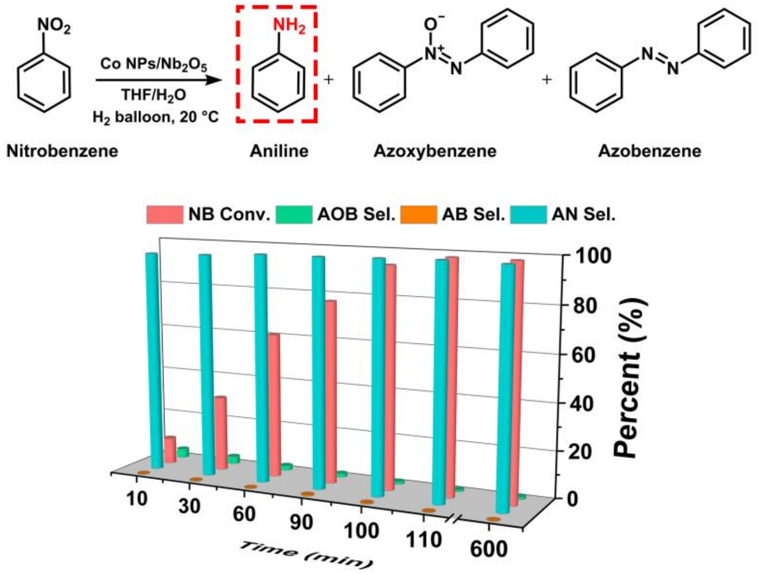

Supplementary Fig. 18 Catalytic performance of Co NPs/Nb_2O_5 in the selective hydrogenation of nitrobenzene to azoxybenzene with solvent.

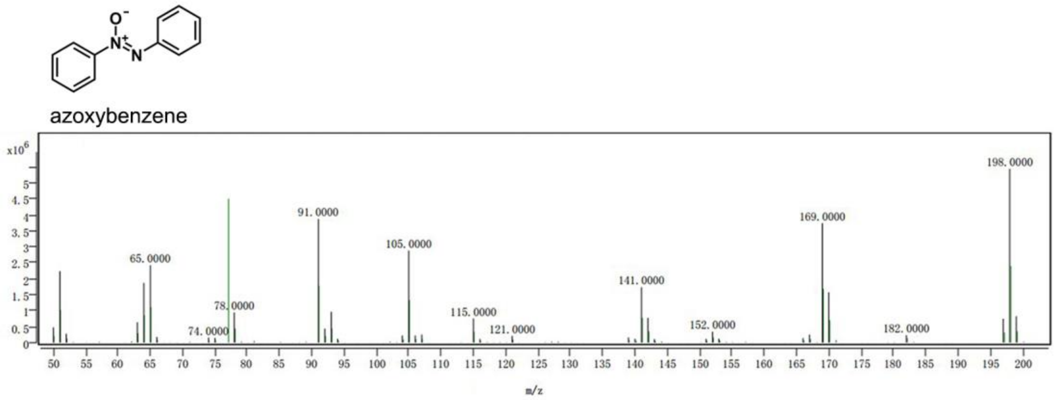

Supplementary Fig. 19 GC-MS analysis of the reaction product of azoxybenzene.

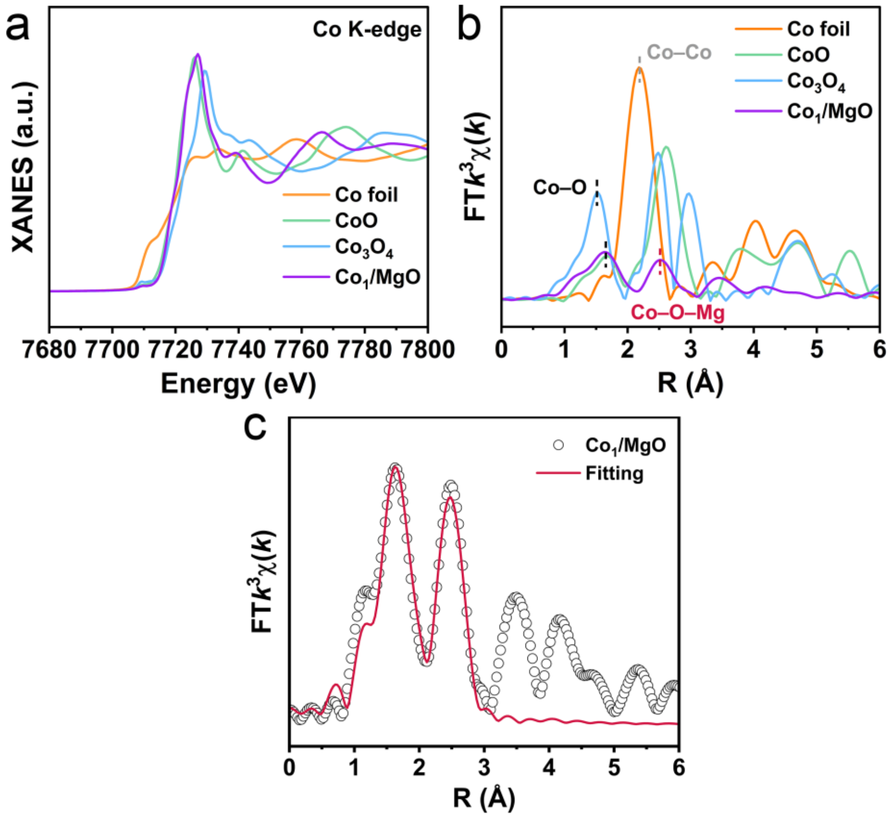

Supplementary Fig. 20 Characterization of the as-prepared Co_1/MgO. (a) XANES spectra at Co K-edge. (b) Fourier transformed k^3-weighted Co K-edge of EXAFS spectra. (c) EXAFS fittings in R space.

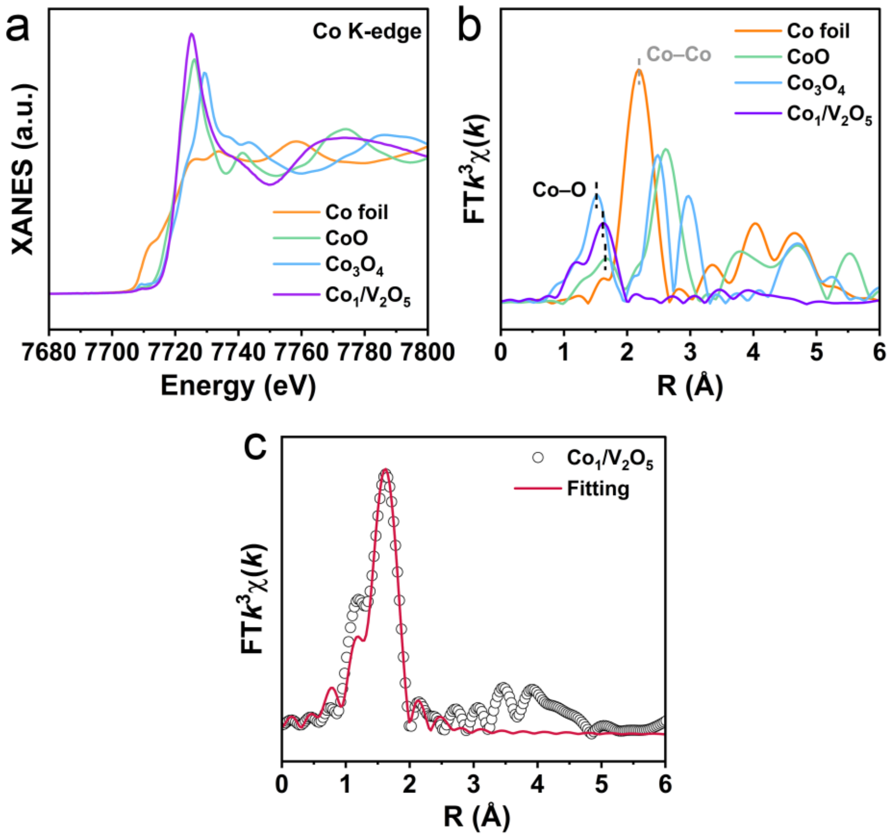

Supplementary Fig. 21 Characterization of the as-prepared Co_1/V_2O_5. (a) XANES spectra at Co K-edge. (b) Fourier transformed k^3-weighted Co K-edge of EXAFS spectra. (c) EXAFS fittings in R space.

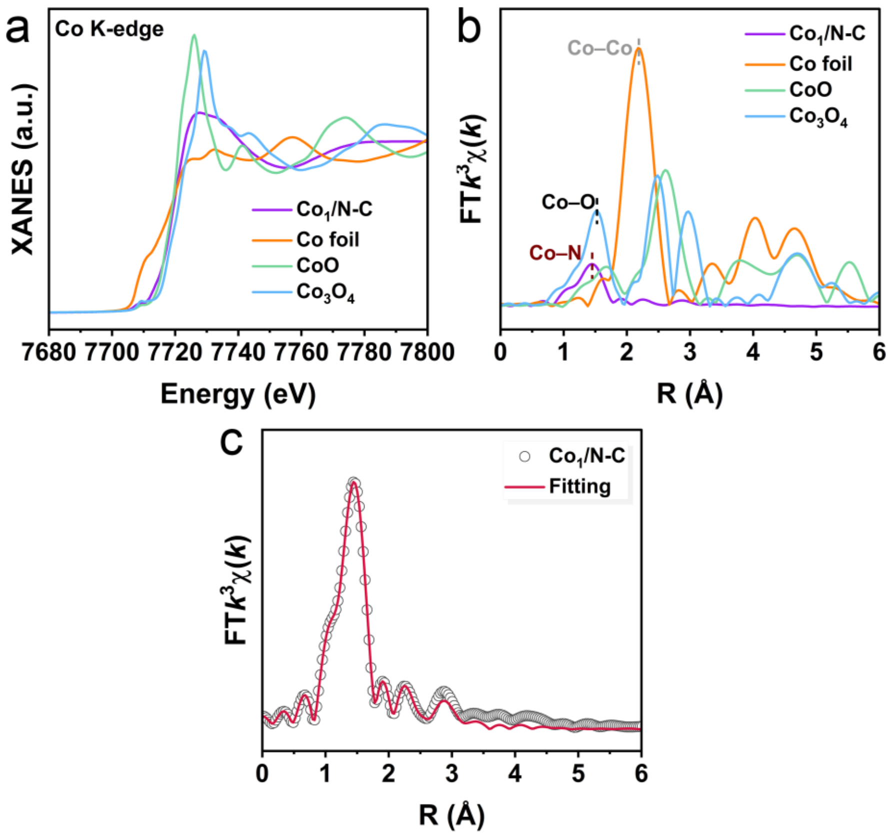

Supplementary Fig. 22 Characterization of the as-prepared Co_1/N-C. (a) XANES spectra at Co K-edge. (b) Fourier transformed k^3-weighted Co K-edge of EXAFS spectra. (c) EXAFS fittings in R space.

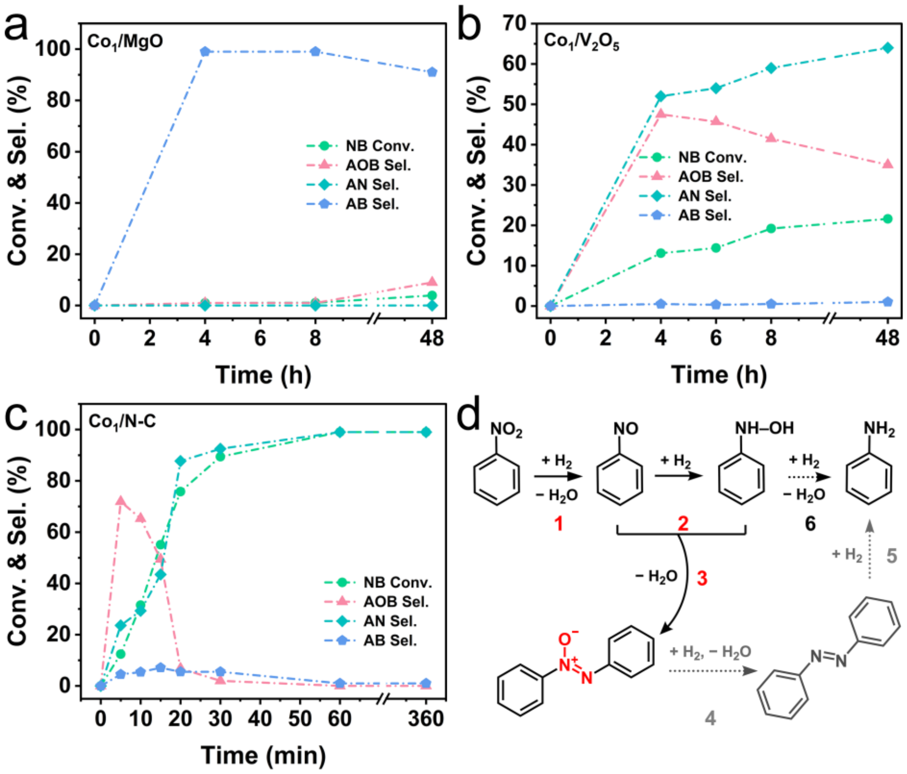

Supplementary Fig. 23 Catalytic performance of (a) Co_1/MgO, (b) Co_1/V_2O_5, and (c) Co_1/N-C in the solvent-free selective hydrogenation of nitrobenzene without solvent. (d) The possible reaction route for Co_1/MgO is 1-2-3-4. The possible reaction route for Co_1/V_2O_5 is 1-2-6, 1-2-3, and 1-2-3-4-5. The possible reaction route for Co_1/N-C is 1-2-3-4-5.

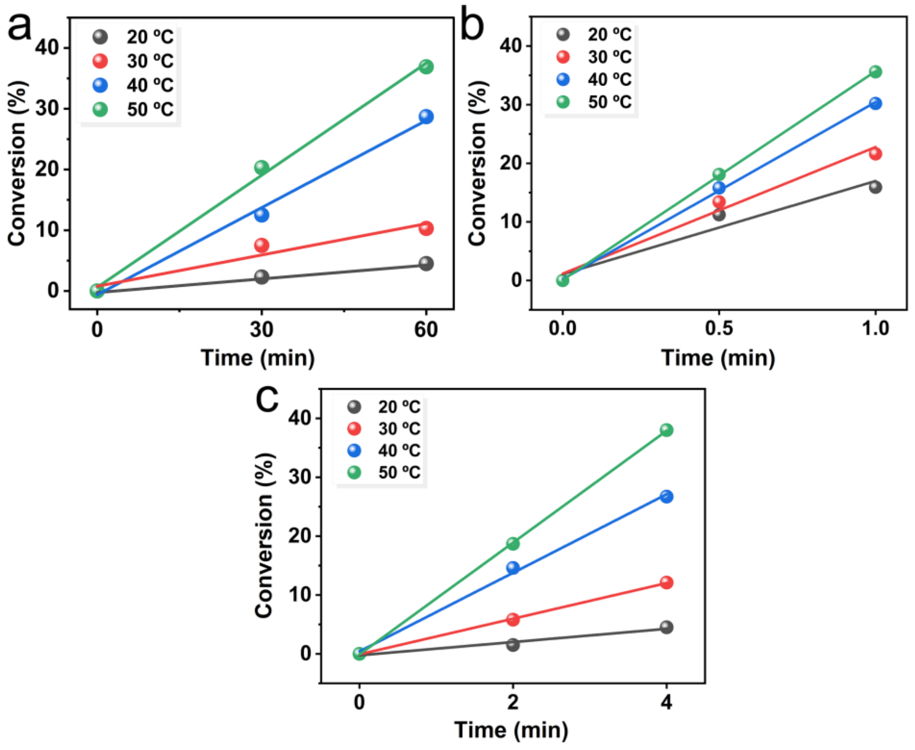

Supplementary Fig. 24 Kinetic results of the solvent-free selective hydrogenation of nitrobenzene to azoxybenzene over (a) Nb_2O_5, (b) Co_1/Nb_2O_5, and (c) Co NPs/ Nb_2O_5 at different temperatures.

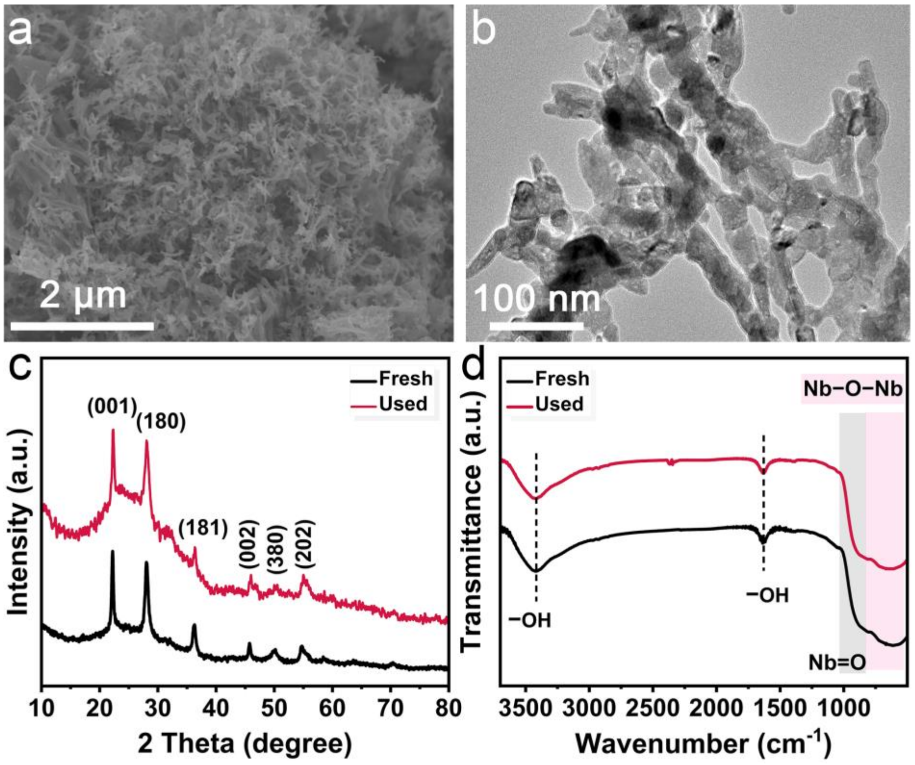

Supplementary Fig. 25 Characterization of the spent Co_1/Nb_2O_5 after recycling tests. (a) SEM image. (b) TEM image. (c) XRD patterns. (d) FT-IR spectra.

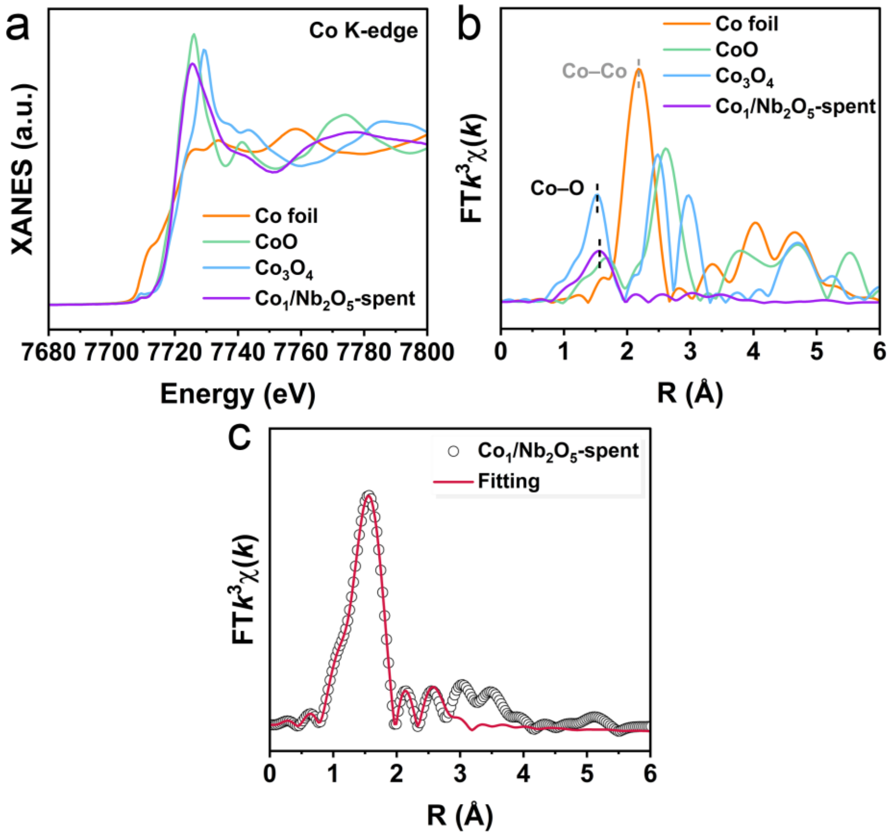

Supplementary Fig. 26 Characterization of the spent Co_1/Nb_2O_5 after recycling tests. (a) XANES spectra at Co K-edge. (b) Fourier transformed k^3-weighted Co K-edge of EXAFS spectra. (c) EXAFS fittings in R space.

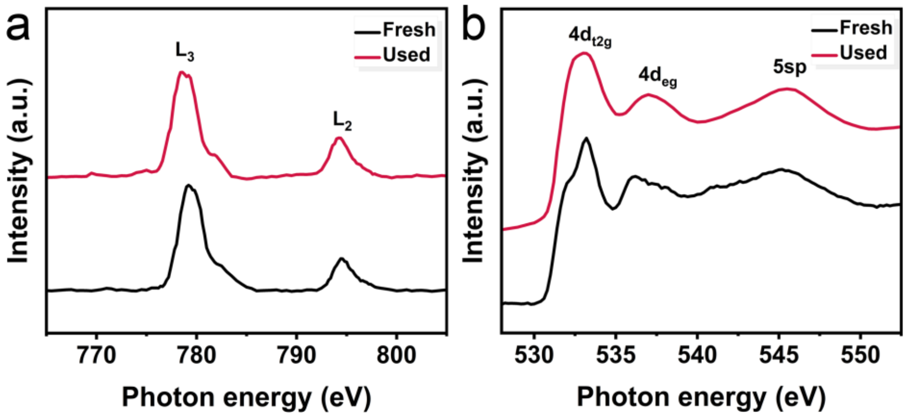

Supplementary Fig. 27 Characterization of the spent Co_1/Nb_2O_5 after recycling tests. (a) Co L-edge and (b) O K-edge spectra.

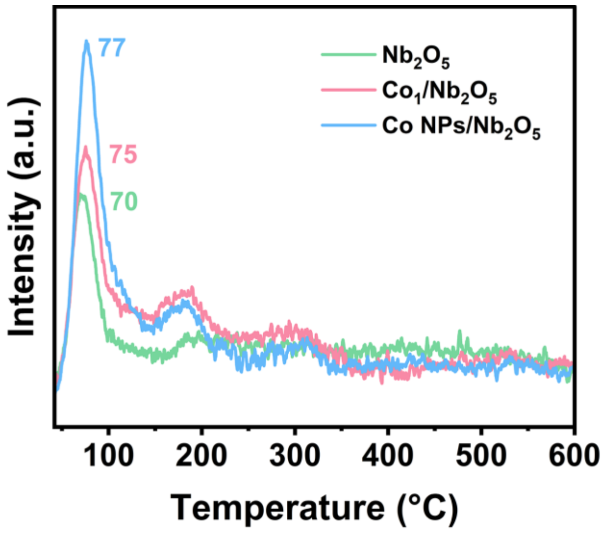

Supplementary Fig. 28 H_2-TPD profiles of Nb_2O_5, Co_1/Nb_2O_5, and Co NPs/Nb_2O_5.

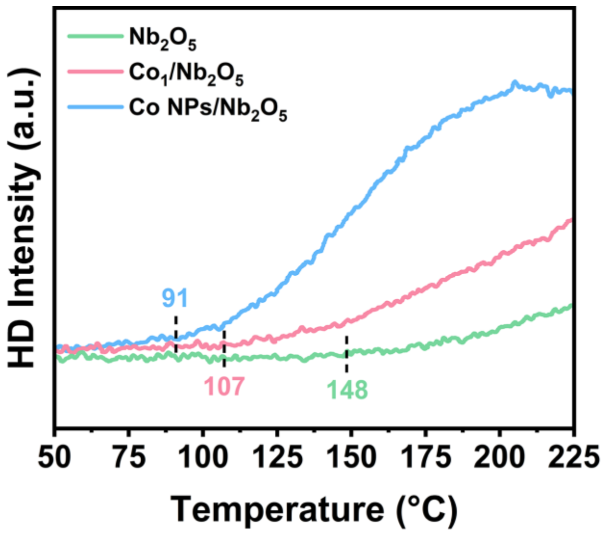

Supplementary Fig. 29 H_2-D_2 exchange results of Nb_2O_5, Co_1/Nb_2O_5, and Co NPs/Nb_2O_5.

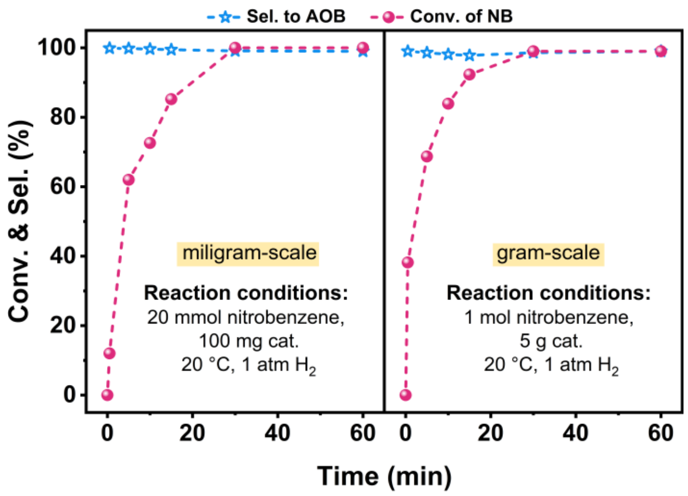

Supplementary Fig. 30 Reaction scale tolerance of Co_1/Nb_2O_5 for solvent-free reaction.

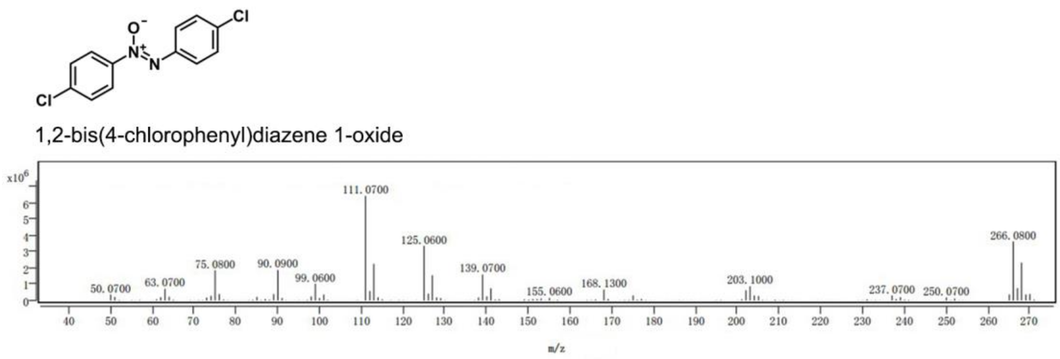

Supplementary Fig. 31 GC-MS analysis of the reaction product of 1,2-bis(4-chlorophenyl)diazene 1-oxide.

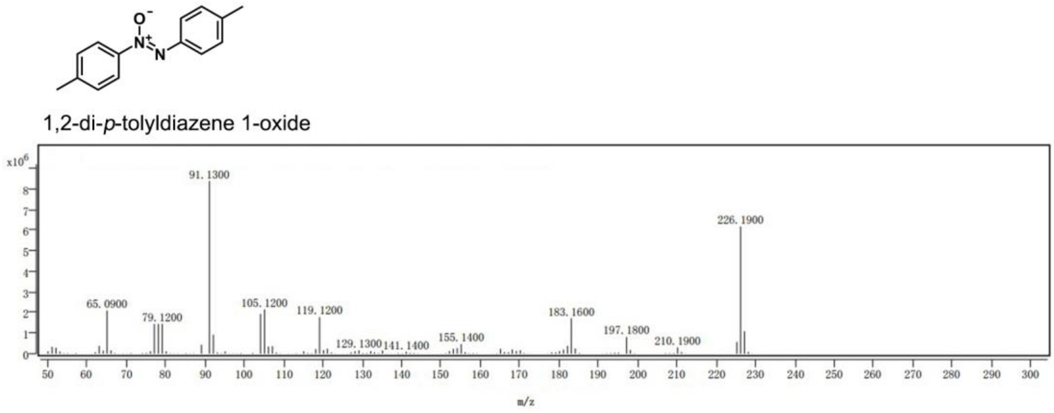

Supplementary Fig. 32 GC-MS analysis of the reaction product of 1,2-di-*p*-tolyldiazene 1-oxide.

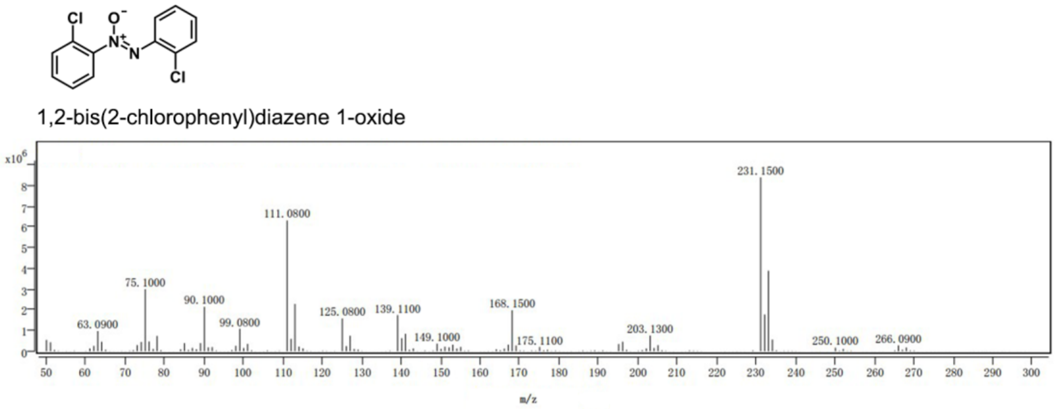

Supplementary Fig. 33 GC-MS analysis of the reaction product of 1,2-bis(2-chlorophenyl)diazene 1-oxide.

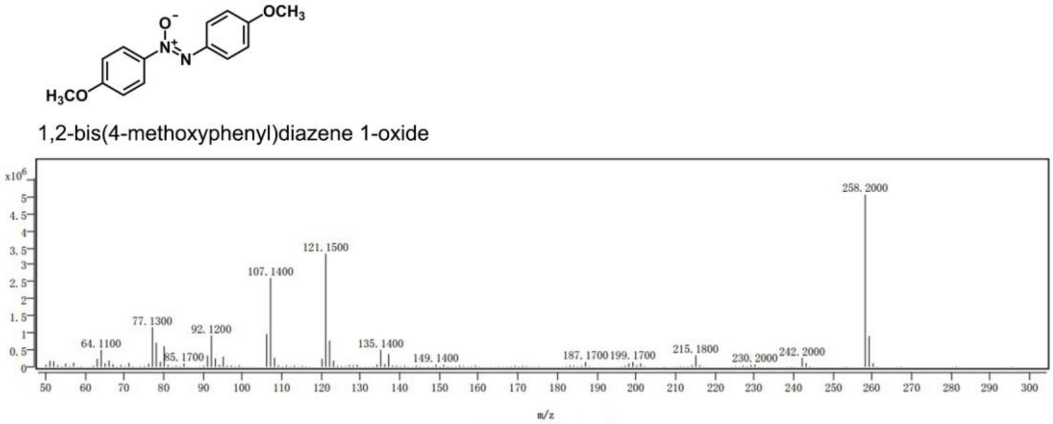

Supplementary Fig. 34 GC-MS analysis of the reaction product of 1,2-bis(4-methoxyphenyl)diazene 1-oxide.

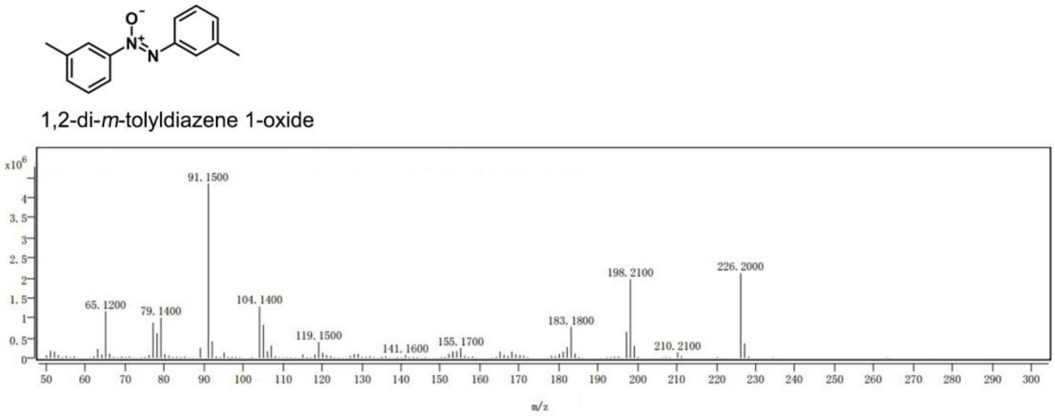

Supplementary Fig. 35 GC-MS analysis of the reaction product of 1,2-di-*m*-tolyldiazene 1-oxide.

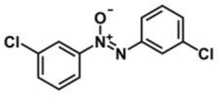

1,2-bis(3-chlorophenyl)diazene 1-oxide

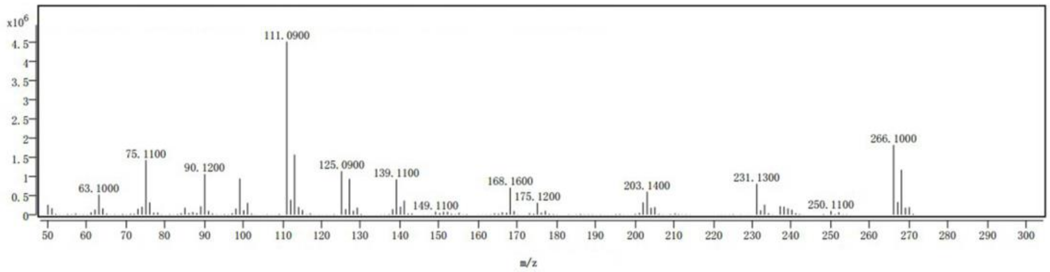

Supplementary Fig. 36 GC-MS analysis of the reaction product of 1,2-bis(3-chlorophenyl)diazene 1-oxide.

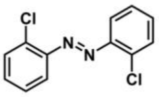

1,2-bis(2-chlorophenyl)diazene

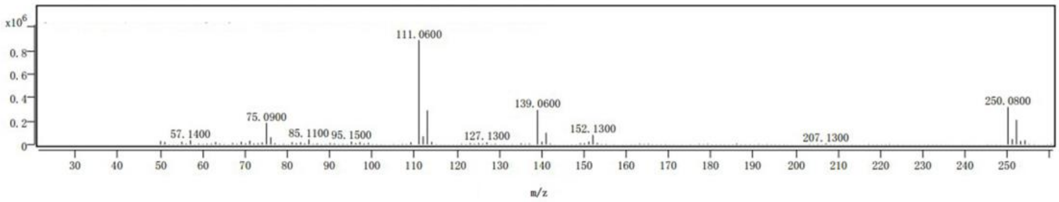

Supplementary Fig. 37 GC-MS analysis of the reaction product of 1,2-bis(2-chlorophenyl)diazene.

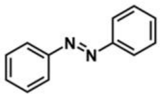

azobenzene

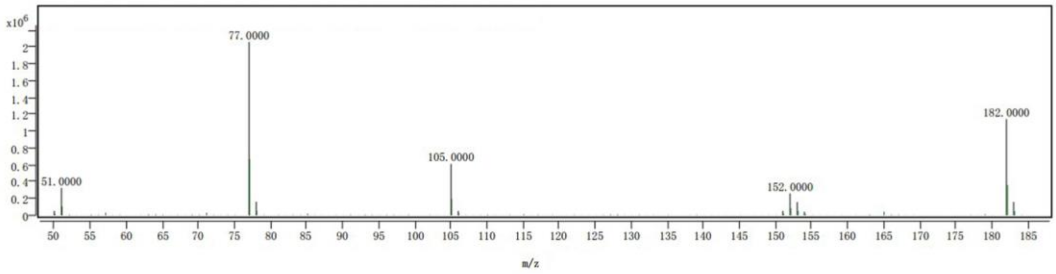

Supplementary Fig. 38 GC-MS analysis of the reaction product of azobenzene.

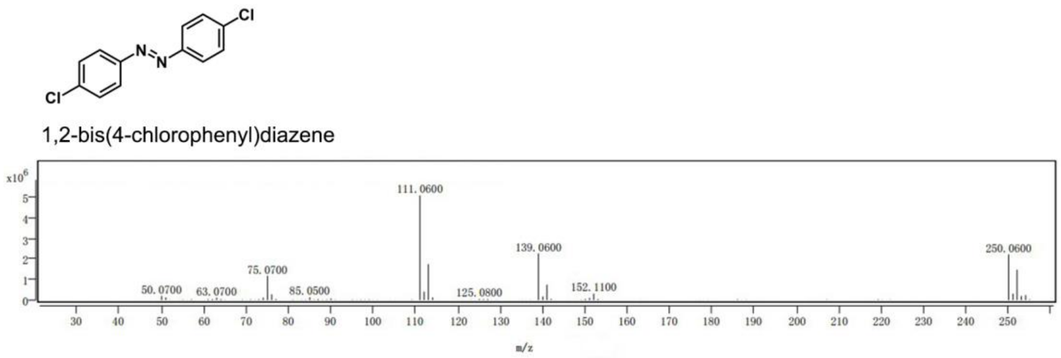

Supplementary Fig. 39 GC-MS analysis of the reaction product of 1,2-bis(4-chlorophenyl)diazene.

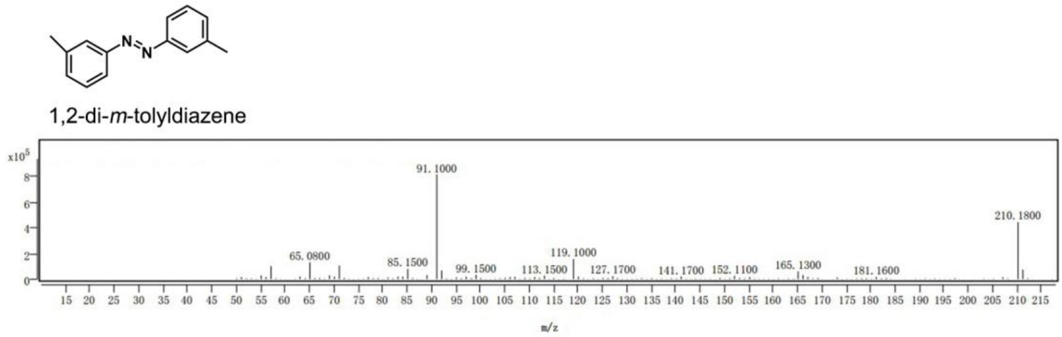

Supplementary Fig. 40 GC-MS analysis of the reaction product of 1,2-di-*m*-tolyldiazene.

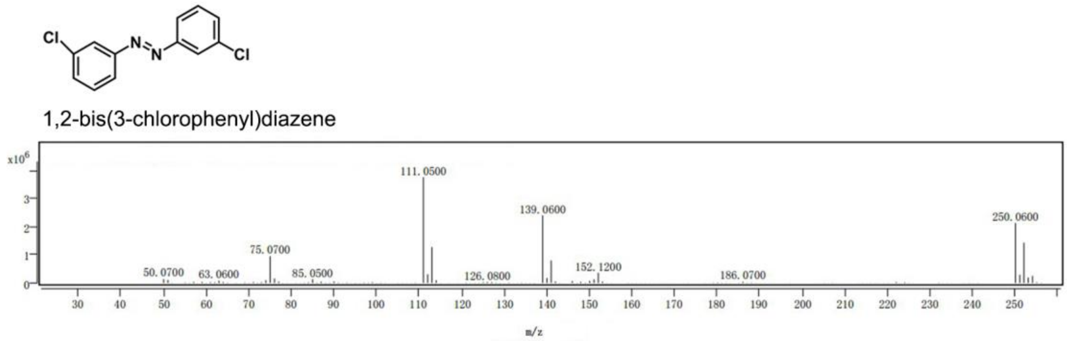

Supplementary Fig. 41 GC-MS analysis of the reaction product of 1,2-bis(3-chlorophenyl)diazene.

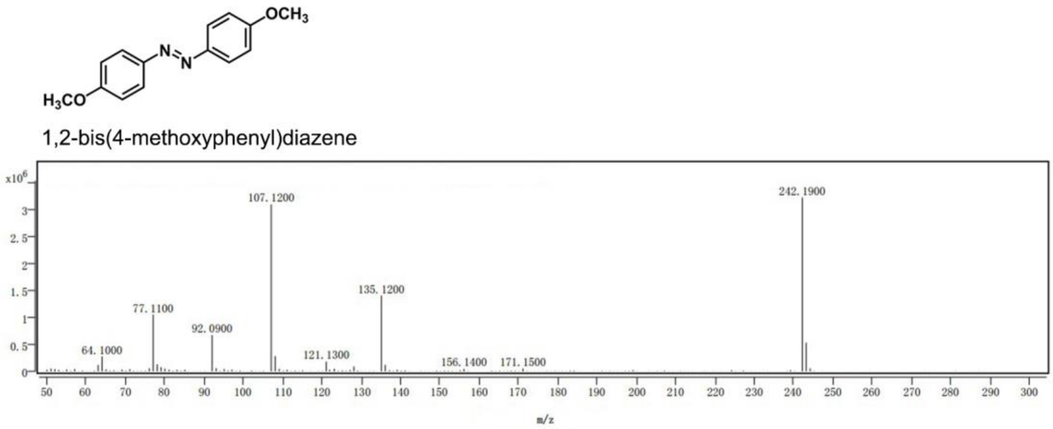

Supplementary Fig. 42 GC-MS analysis of the reaction product of 1,2-bis(4-methoxyphenyl)diazene.

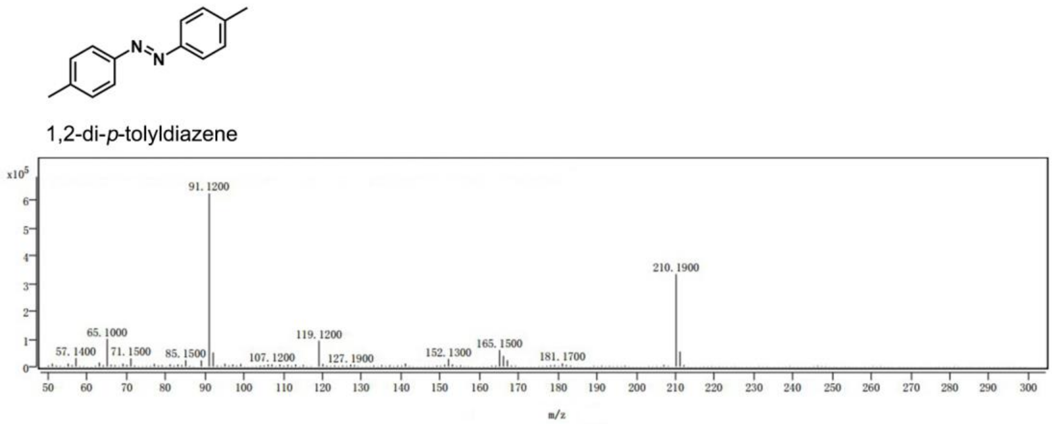

Supplementary Fig. 43 GC-MS analysis of the reaction product of 1,2-di-*p*-tolyldiazene.

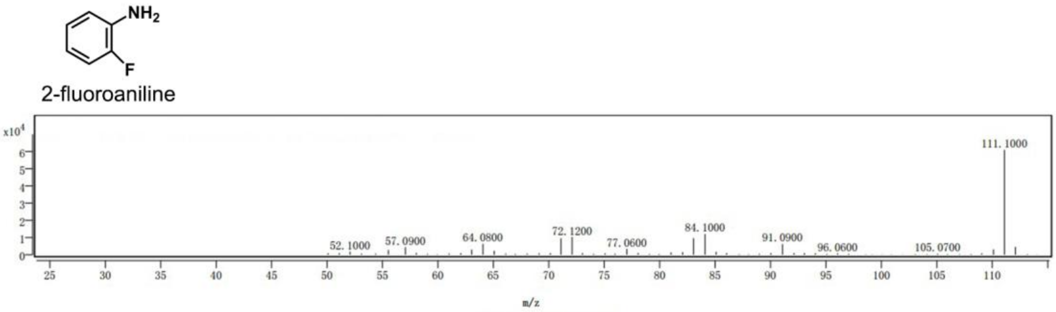

Supplementary Fig. 44 GC-MS analysis of the reaction product of 2-fluoroaniline.

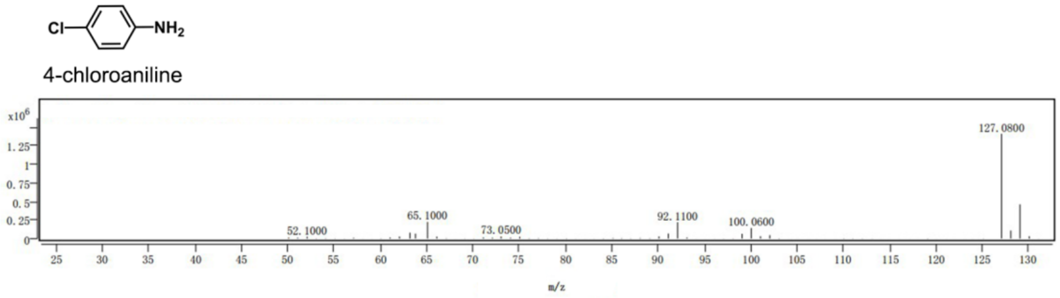

Supplementary Fig. 45 GC-MS analysis of the reaction product of 4-chloroaniline.

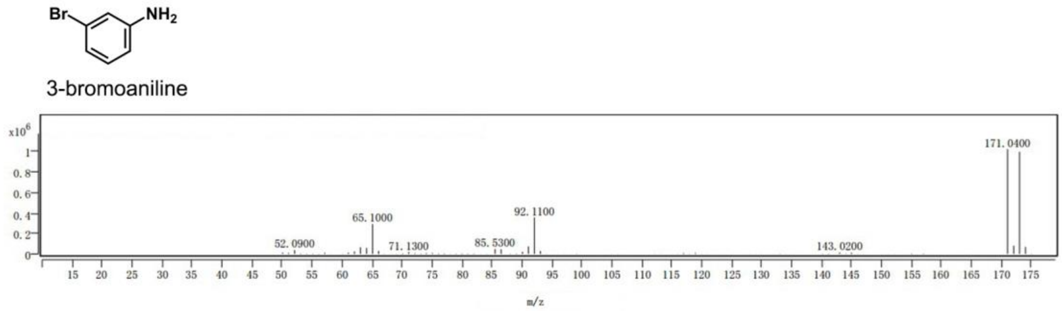

Supplementary Fig. 46 GC-MS analysis of the reaction product of 3-bromoaniline.

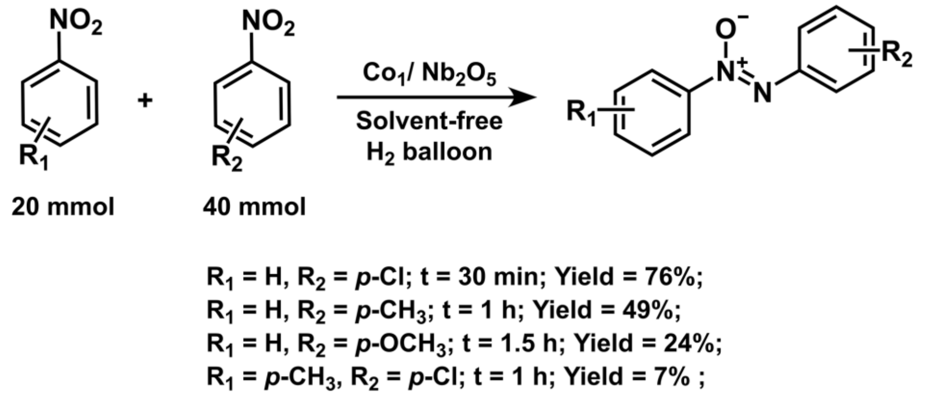

Supplementary Fig. 47 Substrate scope of the solvent-free selective hydrogenation of different nitroaromatics to unsymmetrical azoxy compounds. The yield was based on R_1-$C_6H_4NO_2$.

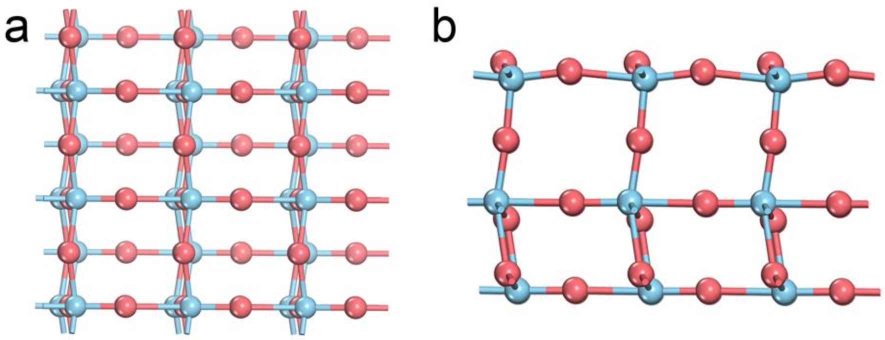

Supplementary Fig. 48 Structural model of Nb_2O_5. (a) Top view. (b) Side view.

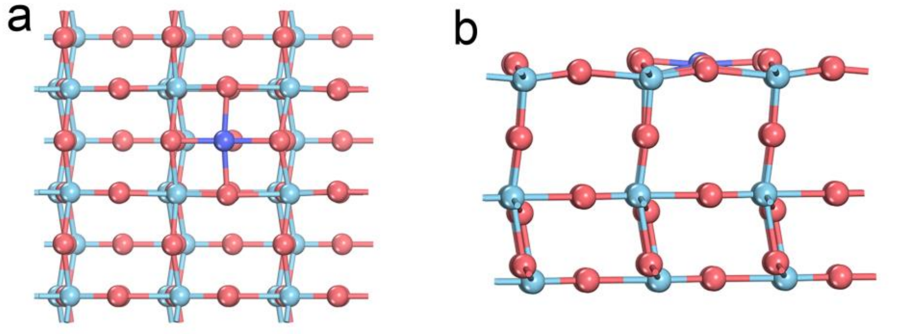

Supplementary Fig. 49 Structural model of Co_1/Nb_2O_5. (a) Top view. (b) Side view.

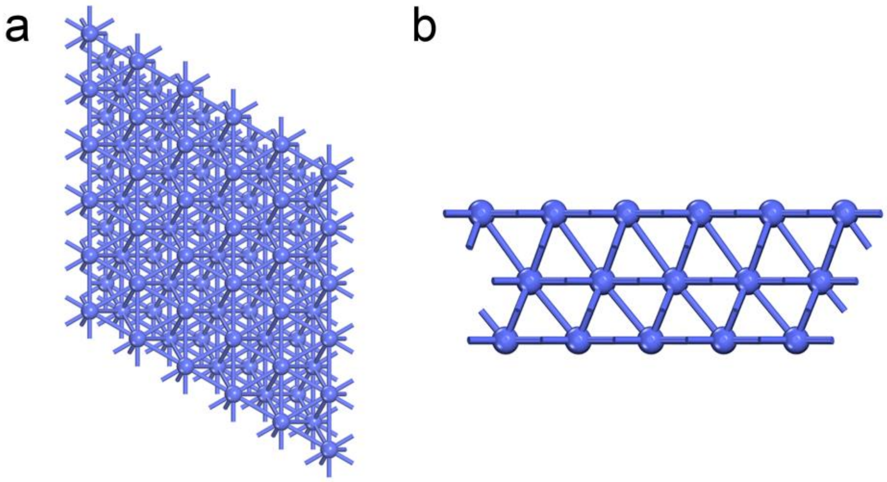

Supplementary Fig. 50 Structural model of Co(111). (a) Top view. (b) Side view.

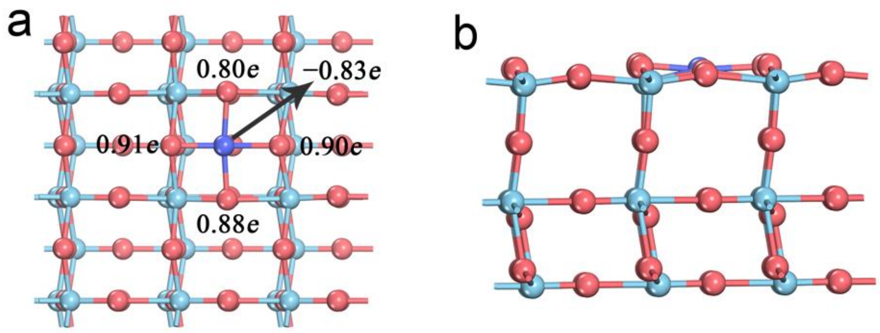

Supplementary Fig. 51 Bader charge of the optimized structure of Co_1/Nb_2O_5. (a) Top view. (b) Side view.

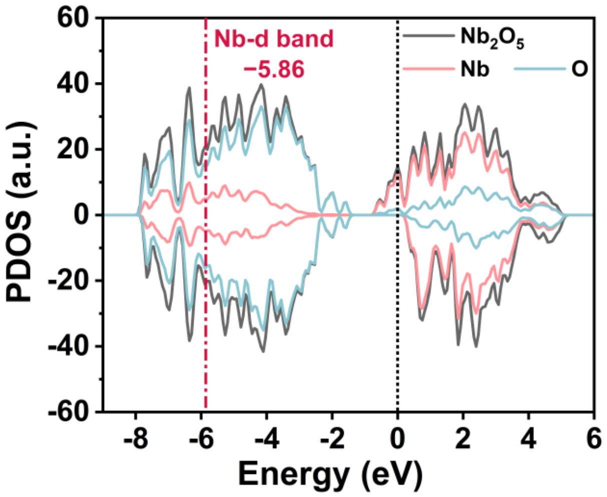

Supplementary Fig. 52 Projected density of states of Nb_2O_5.

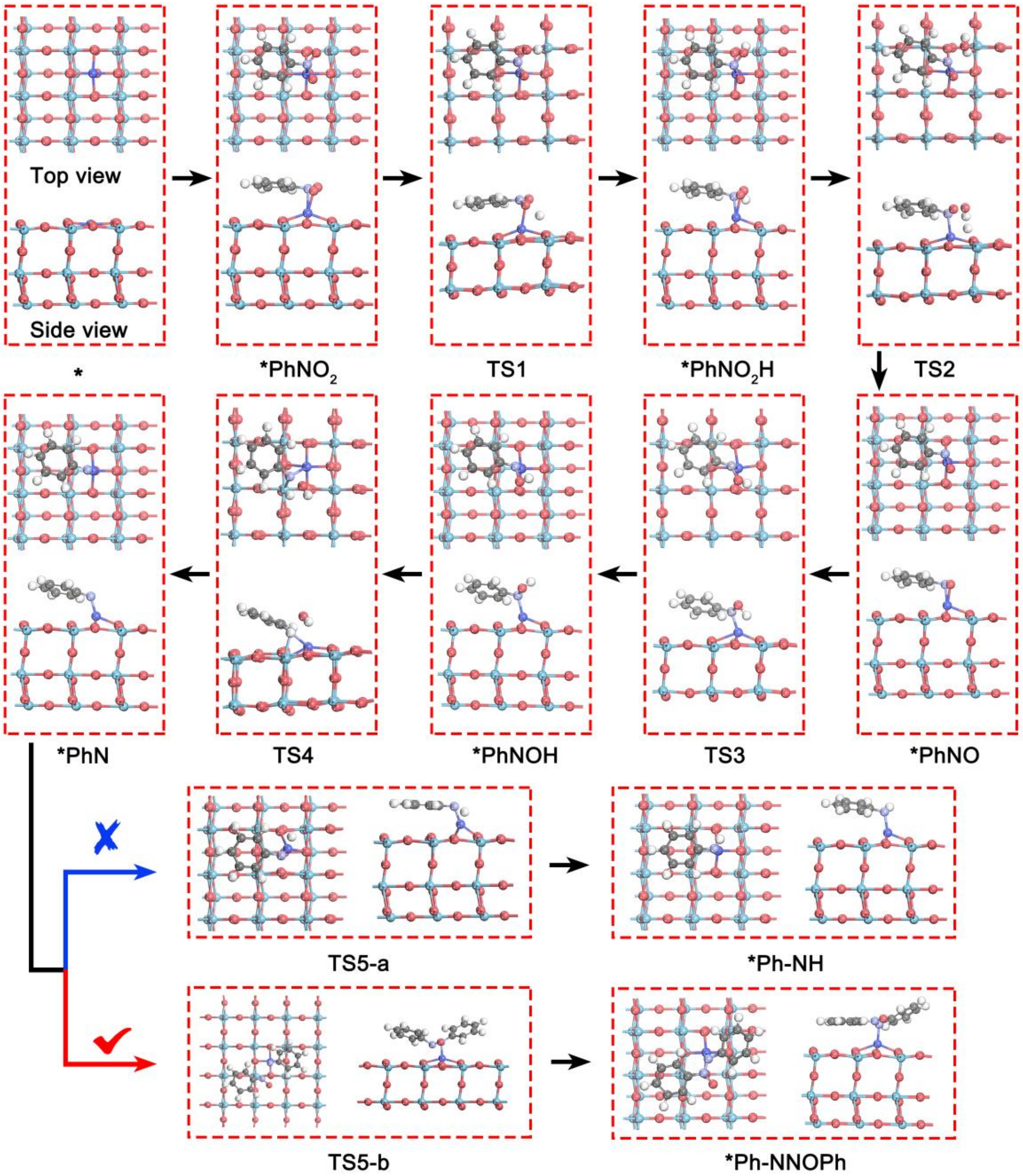

Supplementary Fig. 53 Reaction pathways of the solvent-free selective hydrogenation of nitrobenzene over Co_1/Nb_2O_5 and the corresponding intermediates configurations.

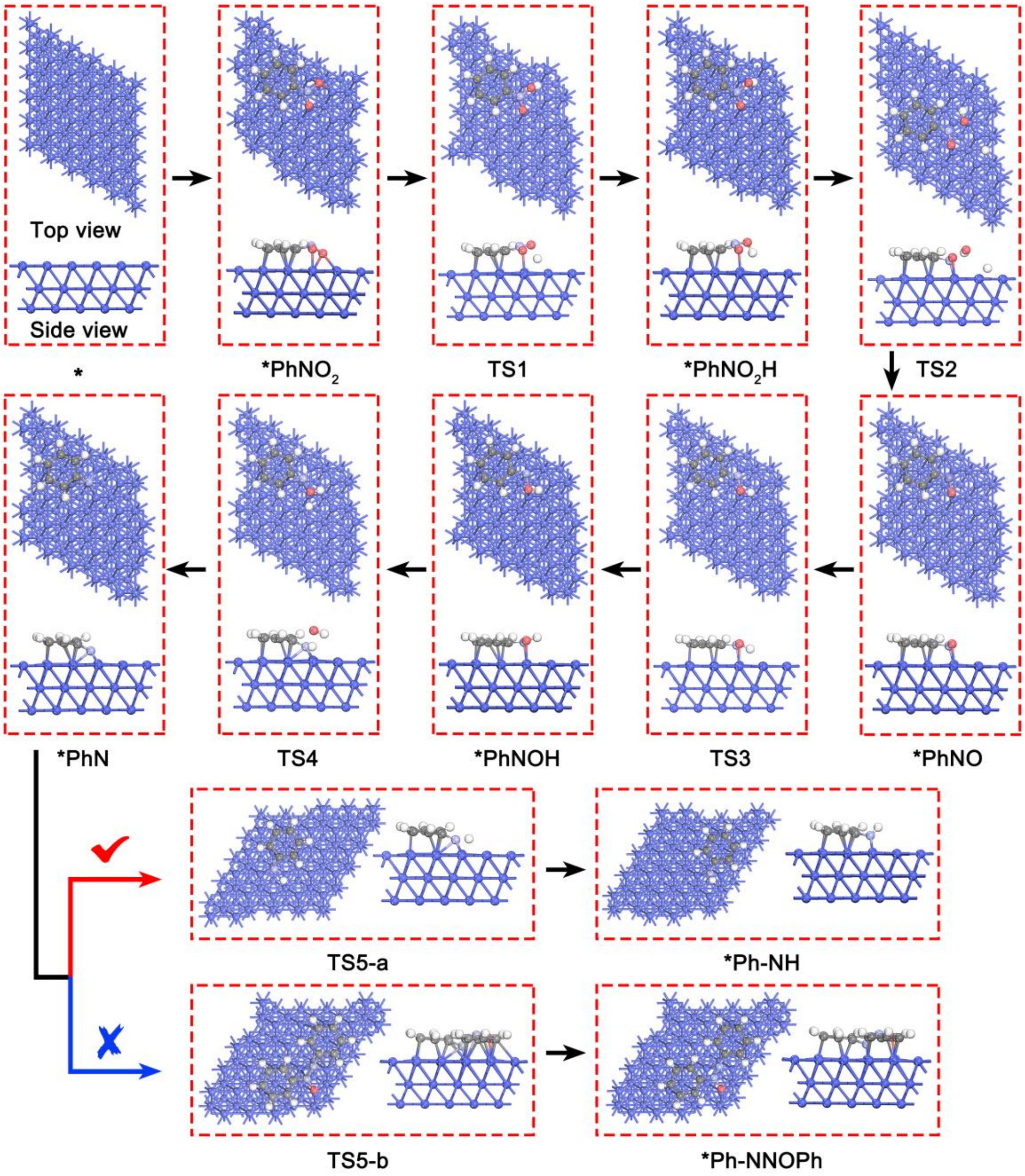

Supplementary Fig. 54 Reaction pathways of the solvent-free selective hydrogenation of nitrobenzene over Co(111) and the corresponding intermediates configurations.

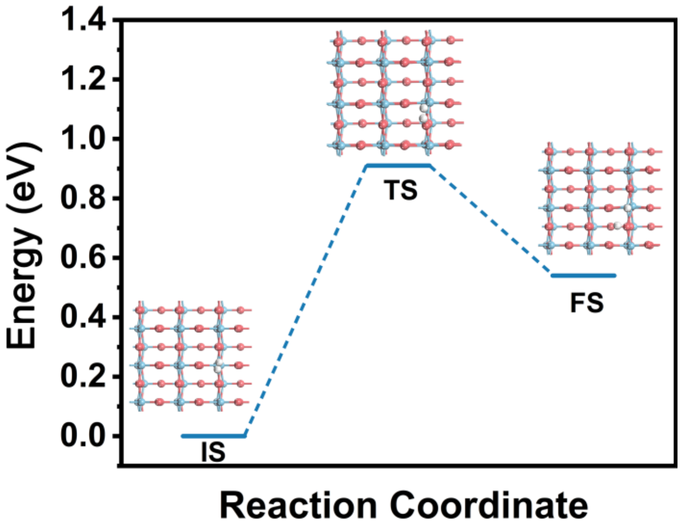

Supplementary Fig. 55 DFT calculation of H_2 dissociation on Nb_2O_5. The inserts are the geometric structures of the initial state (IS), transition state (TS), and final state (FS).

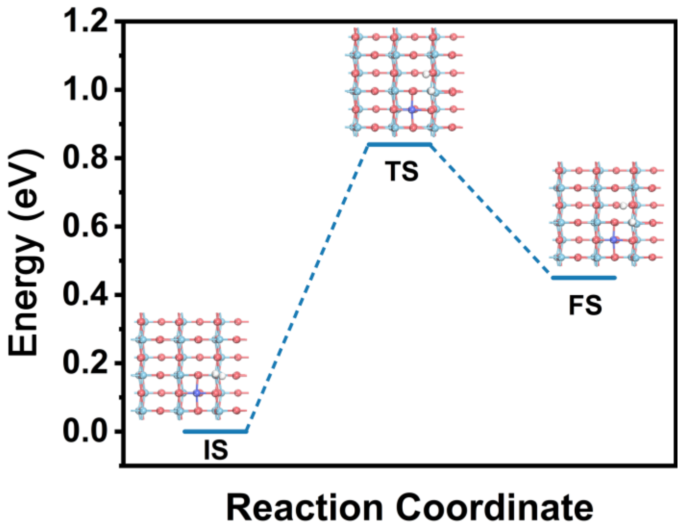

Supplementary Fig. 56 DFT calculation of H_2 dissociation on Co_1/Nb_2O_5. The inserts are the geometric structures of the initial state (IS), transition state (TS), and final state (FS).

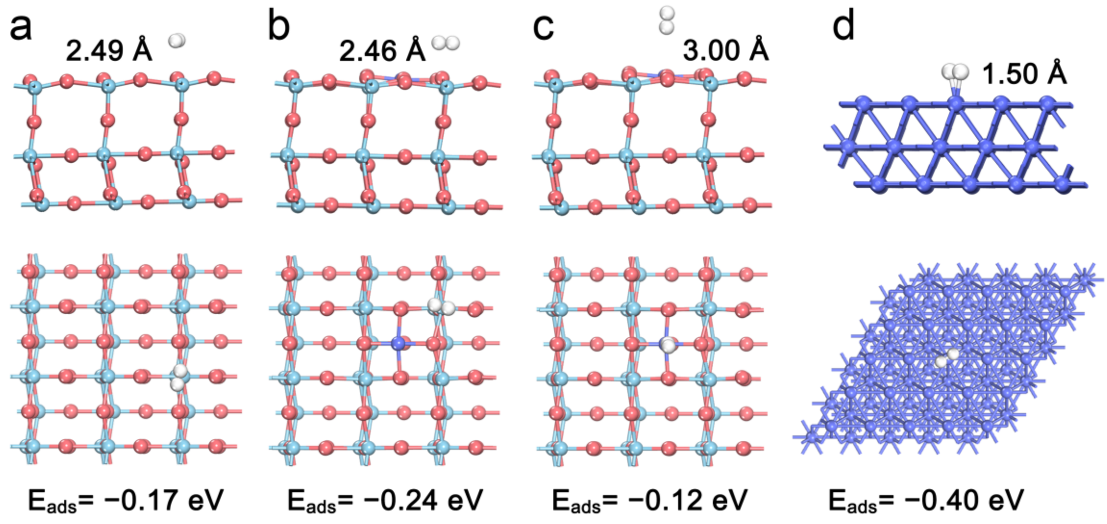

Supplementary Fig. 57 Top and side views of an H_2 molecule adsorbed over (a) Nb_2O_5, (b) Co_1/Nb_2O_5 (Nb site), (c) Co_1/Nb_2O_5 (Co_1 site), and (d) Co(111). The numbers represent the distance between H_2 and the active site. Please note only Co(111) is chemical adsorption, and the rest of cases are physical adsorption.

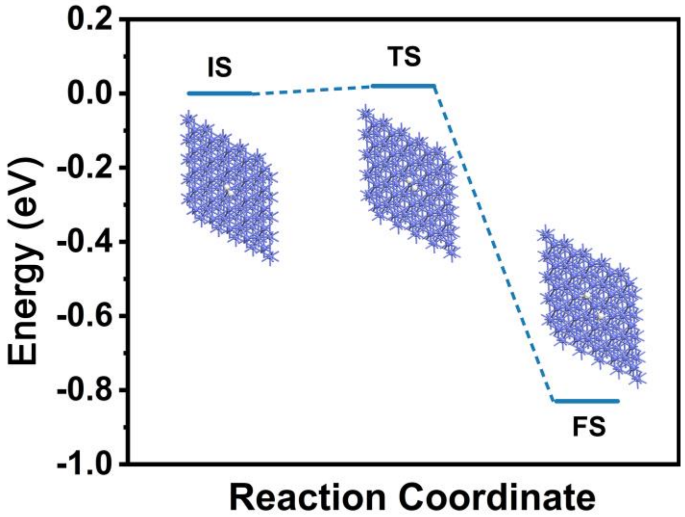

Supplementary Fig. 58 DFT calculation of H_2 dissociation on Co(111). The inserts are the geometric structures of the initial state (IS), transition state (TS), and final state (FS).

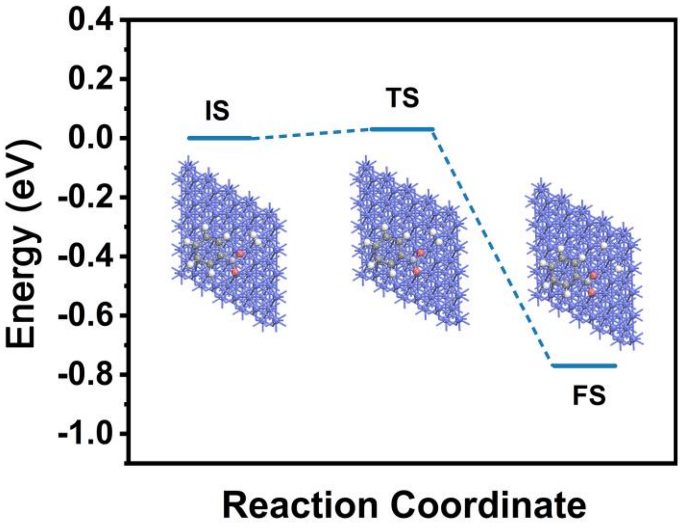

Supplementary Fig. 59 DFT calculation of H_2 dissociation on Co(111) in the presence of *$PhNO_2$. The inserts are the geometric structures of the initial state (IS), transition state (TS), and final state (FS).

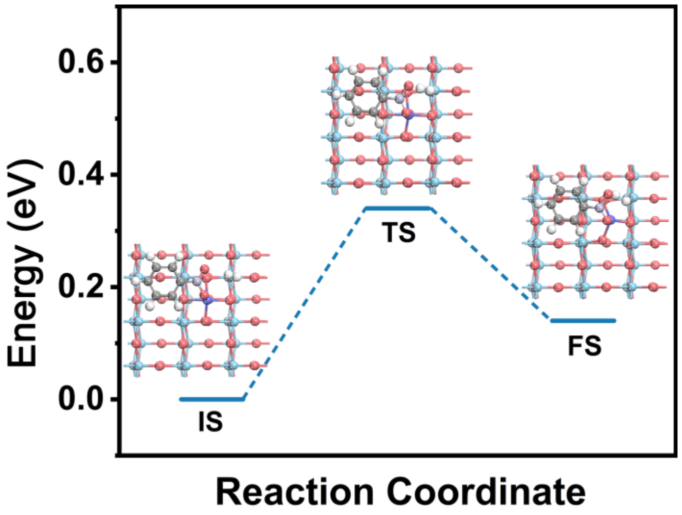

Supplementary Fig. 60 DFT calculation of H_2 dissociation on *$PhNO_2$ of Co_1/Nb_2O_5 via the Eley−Rideal (ER) mechanism. The inserts are the geometric structures of the initial state (IS), transition state (TS), and final state (FS). In this case, H_2 is physically adsorbed above the Nb site near Co_1, and $PhNO_2$ is chemically adsorbed on Co_1; after H_2 dissociation, one H atom ($H^{\delta+}$) transfers to *$PhNO_2$ to form *$PhNO_2H$, and the other one ($H^{\delta-}$) is chemically adsorbed on the Nb site. Please note that the hydrogenation of *$PhNO_2H$ to *PhNO via the $H^{\delta-}$ species adsorbed on the Nb site is kinetically favorable (Fig. 5d); therefore, the cleaving of $H^{\delta+}$ and $H^{\delta-}$ species from Co_1/Nb_2O_5 to the intermediates is favorable.

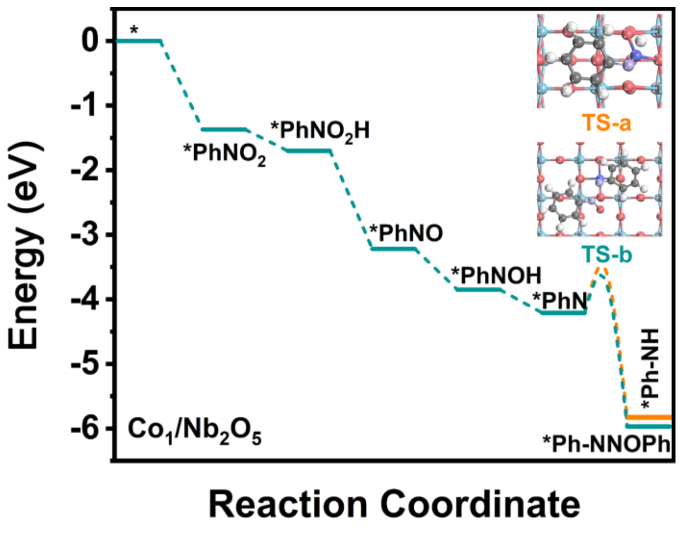

Supplementary Fig. 61 Energy profiles of the selective hydrogenation of nitrobenzene to azoxybenzene with solvent over Co_1/Nb_2O_5.

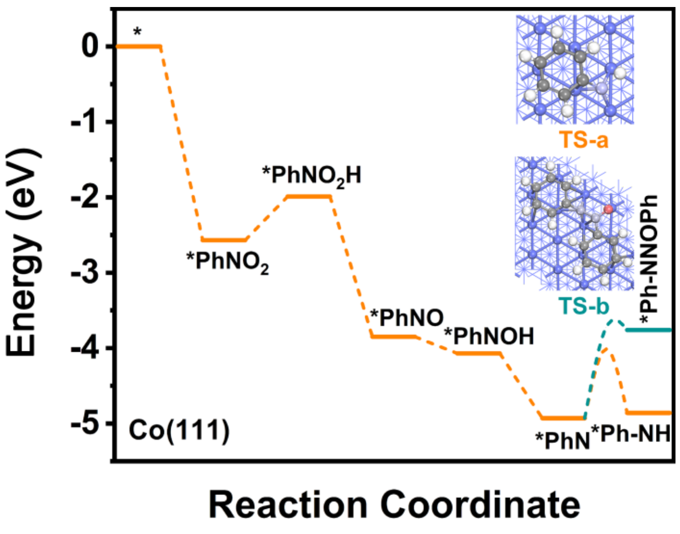

Supplementary Fig. 62 Energy profiles of the selective hydrogenation of nitrobenzene to azoxybenzene with solvent over Co(111).

Supplementary Table 1. Physical properties of samples.

Samples	Co loading[a] (wt %)	Co dispersion[b] (%)		S_{BET}[c] ($m^2 \cdot g^{-1}$)	V_{pore}[d] ($cm^3 \cdot g^{-1}$)	D_{pore}[e] (nm)	H_2 adsorption[f] (μmol/g)
		fresh	used				
Nb_2O_5	–	–	–	49.8	0.16	14.8	38.3
Co_1/Nb_2O_5	0.42	97	93	56.7	0.19	14.5	53.4
Co NPs/Nb_2O_5	5.12	39	33	80.0	0.19	12.3	61.4

[a]Cobalt loading was determined by ICP-AES; [b]Metal dispersion was measured by CO chemisorption; [c]S_{BET} was determined by BET method; [d]Volume of N_2 at P/P_0=0.99; [e]Average pore diameter; [f]Amount of H_2 adsorption derived from H_2-TPD.

Supplementary Table 2. Structural parameters of samples extracted from the EXAFS fitting with reference of Co foil at the Co K-edge. (S_0^2=0.82)

Sample	Path	N	R (Å)	σ^2 (10^{-3}Å^2)	ΔE_0 (eV)	R factor
Co foil	Co-Co	12	2.49	2.7	6.1	0.001
Co_1/Nb_2O_5 (fresh)	Co-O	4.6	2.00	3.0	-4.6	0.001
Co_1/Nb_2O_5 (spent)	Co-O	4.5	2.01	3.0	4.9	0.010
Co_1/MgO	Co-O	4.1	2.11	6.8	2.8	0.011
	Co-O-Mg	5.9	3.01	9.5	-6.3	
Co_1/V_2O_5	Co-O	5.2	2.08	5.1	-0.1	0.010
Co_1/N-C	Co-N	3.8	1.99	5.4	6.3	0.006

N: coordination numbers; *R*: bond distance; σ^2: Debye-Waller factors; ΔE_0: the inner potential correction. *R* factor: goodness of fit.

Supplementary Table 3. The effects of the reaction temperatures and solvents on the selective hydrogenation of nitrobenzene to azoxybenzene over Co_1/Nb_2O_5.

Entry	Solvent	T (ºC)	Time （h）	Conv. (%)	Sel. (%)		
					azoxybenzene	azobenzene	aniline
1[a]	Tetrahydrofuran/H_2O=4:1	20	1.5	>99	>99	–	<1
2[a]	Tetrahydrofuran/H_2O=4:1	60	1	>99	98	–	2
3[a]	Ethanol/H_2O=4:1	20	1.5	78	75.3	1.3	23.4
4[a]	Toluene	20	12	<1	–	–	99
5[b]	Acetonitrile/H_2O=4:1	20	8	>99	13.6	–	86.4
6[a]	Isopropanol	20	10	<1	–	–	99

[a]Reaction conditions: 2 mmol nitrobenzene, 10 mg catalyst, 5 ml solvent, 1 atm H_2.

[b]Reaction conditions: 2 mmol nitrobenzene, 3 ml H_2O_2, 10 mg catalyst, 5 ml solvent, 1 atm H_2.

Supplementary Table 4. Reaction conditions optimization for selective hydrogenation of nitrobenzene to azoxybenzene over Co_1/Nb_2O_5.

Entry	Solvent	Base	Time (h)	Conv. (%)	Sel. (%)		
					azoxybenzene	azobenzene	aniline
1[a]	Tetrahydrofuran/H_2O=4:1	–	1.5	>99	>99	–	<1
2[b]	Ethanol/H_2O=4:1	NaOH	10	<1	–	–	99
3[b]	Isopropanol	NaOH	10	<1	–	–	99
4[a]	Toluene	–	12	<1	–	–	99

[a]Reaction conditions: 2 mmol nitrobenzene, 10 mg catalyst, 5 ml solvent, 20 ºC, 1 atm H_2.
[b]Reaction conditions: 2 mmol nitrobenzene, 10 mg catalyst, 5 ml solvent, 0.06 mmol NaOH, 20 ºC, 1 atm H_2.

Supplementary Table 5. Catalytic performance of different catalysts in the selective hydrogenation of nitrobenzene to azoxybenzene with solvent.

Catalyst	Conv. (%)	Time (min)	Sel. (%)			TOF (h^{-1})
			azoxybenzene	azobenzene	aniline	
Co_1/Nb_2O_5	>99	90	>99	–	<1	11524
Co NPs/Nb_2O_5	>99	110	0.8	0.2	99	4875
Nb_2O_5	11.7	120	39.1	4.8	56.1	–
$Co(NO_3)_2$	19.4	90	60.5	15.8	23.7	429
$CoCl_2$	9.8	90	90.6	3.8	5.6	183
$Co(Ac)_2$	30.3	90	67.3	3.4	29.3	550
CoPc	8.9	90	76.5	9.7	13.8	166

Reaction conditions: 2 mmol nitrobenzene, 10 mg catalyst, 5 ml solvent (THF: H_2O=4:1), 1 atm H_2.

Supplementary Table 6. Catalytic performance comparison between Co_1/Nb_2O_5 and other reported catalysts in the selective hydrogenation of nitrobenzene to azoxybenzene.

Catalyst	M-loading (wt%)	Reaction conditions	Conv. (%)	Sel. (%)	TOF (h^{-1})	Refs.
Solvent-free						This work
Co_1/Nb_2O_5	0.42	1 atm H_2, 20 ºC, 0.5 h	>99	>99	40377	
Co NPs/Nb_2O_5	5.12	1 atm H_2, 20 ºC, 0.5 h	>99	>99 (AN)	6557	
Solvent						
Nb_2O_5	–	1 atm H_2, 20 ºC, 2 h, THF:H_2O (v:v=4:1)	11.7	39.1	–	
Co_1/Nb_2O_5	0.42	1 atm H_2, 20 ºC, 1.5 h, THF:H_2O (v:v=4:1)	>99	99	11524	
Co NPs/Nb_2O_5	5.12	1 atm H_2, 20 ºC, 110 min,THF:H_2O (v:v=4:1)	>99	>99 (AN)	4875	
Co_1/G	0.4/0.8/1.3 /2.0/2.5	1 atm Ar, $NaBH_4$, THF:H_2O (v:v=8:2), 25 ºC, 1h	~100%	Yield: ~90%	~1188	13
Ni/CeO_2	1.94	1 atm Ar, 80 ºC, ethanol, p-xylene, 80 min, $N_2H_4\cdot H_2O$	99	Yield: 92	36.7	14
Ru-SAs/CeO_2	1.6	3 MPa H_2, 100 ºC isopropanol, 8 h	100	Yield: 65	–	15
h-Ni_1N_4/NC	3.3	Ar, NaOH, isopropyl alcohol, n-dodecane, 50 ºC, 3.5 h	99.2	Yield: 95.6	33.4	16
l-Ni_1N_5/NC	1.5	Ar, NaOH, isopropyl alcohol, n-dodecane, 50 ºC, 3.5 h	38.8	Yield: 33.1	13.5	
5NiO-Au/TiO_2	0.32	Ar, KOH, isopropyl alcohol, 60 ºC, 5 h	100	Yield: 98.4	–	17
Ni/G nanocomposite	–	$N_2H_4\cdot H_2O$, ethanol, 25 ºC, 2 h	100	100	18.1	18
Au/meso-CeO_2	0.5	1 atm N_2, 2-propanol, H_2O, KOH, 30 ºC, 5 h	100	Yield: >99	–	19
Au@CeO_2 CSNs	11.2	1 atm Ar, 2-propanol, KOH, 50 ºC, 11 h	98	Yield: 92	–	20
Au@CeO_2[P]	$5*10^{-6}$ mol	$NaBH_4$, ethanol, 70 ºC, 1 h	91	89.3	363.2	21

AuNP@PPh_2-PEGPIILP	0.005 mol %	$NaBH_4$, ethanol, 25 ºC, 150 min	100	100	37000	22
Ir NSs	0.25 mol%	1 atm H_2, ethanol, n-butylamine, 25 ºC, 1.5 h	100	88.5	399.4	
Rh NSs	0.25 mol%	1 atm H_2, ethanol, n-butylamine, 25 ºC, 4 h	100	84.7	186.5	23
Ir–Rh NSs	0.25 mol%	1 atm H_2, ethanol, n-butylamine, 25 ºC, 2.33 h	100	86.1	279.4	
Ni-$TiO_2^{EG–W–H}$	2.6	H_2O, $N_2H_4 \cdot H_2O$, 25 ºC, 12 h	89	Yield: 92	TON: 1008	24
Ni@C+CeO_2	5	10 bar H_2, toluene, 120 ºC, 50 min	100	94.1	>5000	25
Ni-PHI	1	$NaBH_4$, ethanol, 50 ºC, 3 h	99	Yield: 98	435	26
Rod-CeO_2	–	5 bar N_2, anhydrous toluene, 150 ºC, 4 h	100	90.4	–	27

Supplementary Table 7. The recycling results of Co_1/Nb_2O_5 in the solvent-free selective hydrogenation of nitrobenzene to azoxybenzene.

Cycle number	Conv. (%)	Sel. (%)
1	61.8	>99
2	61.2	>99
3	60.8	>99
4	61.3	>99
5	60.9	>99
6	61.5	>99
7	60.3	>99
8	60.9	>99
9	60.7	>99
10	60.5	>99

Reaction conditions: 2 mmol nitrobenzene, 10 mg catalyst, 5 min, 1 atm H_2.

Supplementary References

1. Fang, H. et al. Dispersed surface Ru ensembles on MgO(111) for catalytic ammonia decomposition. *Nat. Commun.* **14**, 647 (2023).
2. Li, Z. et al. A heterogeneous single atom cobalt catalyst for highly efficient acceptorless dehydrogenative coupling reactions. *Small* **19**, 2207941 (2023).
3. Kresse, G. & Furthmüller, J. Efficient iterative schemes for *ab initio* total-energy calculations using a plane-wave basis set. *Phys. Rev. B* **54**, 11169−11186 (1996).
4. Kresse, G. & Joubert, D. From ultrasoft pseudopotentials to the projector augmented-wave method. *Phys. Rev. B* **59**, 1758−1775 (1999).
5. Blöchl, P. E. Projector augmented-wave method. *Phys. Rev. B* **50**, 17953−17979 (1994).
6. Perdew, J. P., Burke, K. & Ernzerhof, M. Generalized gradient approximation made simple. *Phys. Rev. Lett.* **77**, 3865–3868 (1996).
7. Shang, C. & Liu, Z.-P. Constrained broyden minimization combined with the dimer method for locating transition state of complex reactions. *J. Chem. Theory Comput.* **6**, 1136–1144 (2010).
8. Zhang, X.-J., Shang, C. & Liu, Z.-P. Double-ended surface walking method for pathway building and transition state location of complex reactions. *J. Chem. Theory Comput.* **9**, 5745–5753 (2013).
9. Mathew, K., Sundararaman, R., Letchworth-Weaver, K., Arias, T. A. & Hennig, R. G. Implicit solvation model for density-functional study of nanocrystal surfaces and reaction pathways. *J. Chem. Phys.* **140**, 084106 (2014).
10. Justice, M.-C., Justice, J.-C. & Kay, R. L. A conductance investigation of HCl in aqueous solutions of tetrahydrofuran, dimethoxyethane and dioxane. *J. Solution Chem.* **19**, 1211–1224 (1990).
11. Grimme, S. Semiempirical GGA-type density functional constructed with a long-range dispersion correction. *J. Comput. Chem.* **27**, 1787–1799 (2006).
12. Jain, A. et al. Formation enthalpies by mixing GGA and GGA + *U* calculations. *Phys. Rev. B* **84**, 045115 (2011).
13. Yan, H. et al. Atomic engineering of high-density isolated Co atoms on graphene with proximal-atom controlled reaction selectivity. *Nat. Commun.* **9**, 3197 (2018).
14. Hou, T. et al. Peculiar hydrogenation reactivity of $Ni–Ni^{\delta+}$ clusters stabilized by ceria in reducing nitrobenzene to azoxybenzene. *J. Catal.* **353**, 107–115 (2017).
15. Wu, B. et al. Ru single atoms for efficient chemoselective hydrogenation of nitrobenzene to azoxybenzene. *Green Chem.* **23**, 4753–4761 (2021).
16. Zhang, T. et al. Selective transfer hydrogenation coupling of nitroaromatics to azoxy/azo compounds by electron-enriched single $Ni-N_4$ sites on mesoporous N-doped carbon. *Chem. Eng. J.* **443**, 136416 (2022).
17. Zhao, J.-X. et al. Selectivity regulation in Au-catalyzed nitroaromatic hydrogenation by anchoring single-site metal oxide promoters. *ACS Catal.* **10**, 2837–2844 (2020).
18. Pahalagedara, M. N. et al. Room temperature selective reduction of nitrobenzene to azoxybenzene over magnetically separable urchin-like Ni/graphene nanocomposites. *J. Catal.* **336**, 41–48 (2016).

19. Liu, X. et al. Mild, selective and switchable transfer reduction of nitroarenes catalyzed by supported gold nanoparticles. *Catal. Sci. Technol.* **3**, 3200–3206 (2013).
20. Li, J. et al. Investigating the hybrid-structure-effect of CeO_2-encapsulated Au nanostructures on the transfer coupling of nitrobenzene. *Adv. Mater.* **30**, 1704416 (2018).
21. Chong, H. et al. Design of an ultrasmall Au nanocluster-CeO_2 mesoporous nanocomposite catalyst for nitrobenzene reduction. *Nanoscale* **5**, 7622–7628 (2013).
22. Doherty, S. et al. Highly selective and solvent-dependent reduction of nitrobenzene to N-phenylhydroxylamine, azoxybenzene, and aniline catalyzed by phosphino-modified polymer immobilized ionic liquid-stabilized AuNPs. *ACS Catal.* **9**, 4777–4791 (2019).
23. Zhang, Z.-P. et al. Free-standing iridium and rhodium-based hierarchically-coiled ultrathin nanosheets for highly selective reduction of nitrobenzene to azoxybenzene under ambient conditions. *Nanoscale* **8**, 15744–15752 (2016).
24. Shukla, A. et al. Room temperature selective reduction of nitroarenes to azoxy compounds over Ni-TiO_2 catalyst. *Mol. Catal.* **490**, 110943 (2020).
25. Liu, L., Concepción, P. & Corma, A. Modulating the catalytic behavior of non-noble metal nanoparticles by inter-particle interaction for chemoselective hydrogenation of nitroarenes into corresponding azoxy or azo compounds. *J. Catal.* **369**, 312–323 (2019).
26. da Silva, M. A. R. et al. Simple and straightforward method to prepare highly dispersed Ni sites for selective nitrobenzene coupling to azo/azoxy compounds. *Chem. Eng. J.* **460**, 141068 (2023).
27. Zhou, X. et al. Enhancing nitrobenzene reduction to azoxybenzene by regulating the O-vacancy defects over rationally tailored CeO_2 nanocrystals. *Appl. Surf. Sci.* **572**, 151343 (2022).
